# Mapping AI startup investment and innovation in healthcare using a five-tier AI systems complexity framework

**DOI:** 10.1038/s41746-026-02595-5

**Published:** 2026-04-14

**Authors:** Ahmed Zahlan, Pek Hooi Soh, Bart Clarysse

**Affiliations:** 1 Global Business School for Health, University College of London, London, UK; 2 Beedie School of Business, Simon Fraser University, Vancouver, BC Canada; 3 Department of Management, Technology, and Economics, ETH Zurich, Zurich, Switzerland

**Keywords:** Business and industry, Health care, Mathematics and computing, Scientific community, Social sciences

## Abstract

Artificial Intelligence (AI) is reshaping healthcare through advances in diagnostics, treatment, and operations, yet the startup ecosystem driving this transformation remains underexplored. Analyzing 3,807 AI health startups founded between 2010 and 2024, this study applies a five-tier framework of AI systems complexity to classify ventures by medical domain, AI systems level, funding, geography, and team composition. Nearly two-thirds of AI investments focus on clinical decision support, drug discovery, and diagnostics, domains associated with higher-complexity deep-learning systems, while areas such as mental health, public health, and rehabilitation attract less AI venture capital, reflecting scalability and data limitations rather than a lack of need. Startups remain concentrated in high-income countries, and founding teams are predominantly technical and business-oriented, with limited clinical representation and gender diversity. By linking these empirical patterns to the five-tier framework, we show how AI systems complexity shapes innovation pathways, offering a foundation for more equitable, evidence-driven digital-medicine ecosystems.

## Introduction

Artificial intelligence (AI) and Machine Learning (ML) enabled technologies have significantly advanced healthcare, demonstrating notable improvements in diagnostics^1,2^, treatment planning^3^, and operational efficiency^4,5^. These innovations are projected to save up to $360 billion annually in the U.S. healthcare system^6^, based on 2019 spending levels, assuming broad AI adoption. AI has the potential to restore humanity to healthcare by automating routine tasks and allowing clinicians to devote more time to patient care^7^. In this era of AI-enabled healthcare, the aspiration is not only to improve our healthcare systems but to support physicians in delivering truly patient-centered medicine.

Despite growing optimism, the integration of AI into medical practice faces persistent challenges related to clinical effectiveness, safety, and validation^8–10^. Studies have shown variability in AI performance across different clinical tasks. For example, ChatGPT-3.5 and GPT-4 demonstrated inconsistent accuracy in managing Parkinson’s disease^11^, and AI tools designed to support radiologists have produced mixed results in diagnostic settings^12^. As Lee et al.^13^, caution, GPT-4 is prone to “hallucinations,” or the generation of plausible yet incorrect information, which can lead to subtle but potentially dangerous diagnostic errors, underscoring the need for vigilant human oversight. Concerns around transparency and regulatory rigor further complicate adoption: many AI-enabled devices are approved through the FDA’s 510(k) clearance pathway, which emphasizes similarity to existing technologies rather than rigorous, independent validation^14^. While this pathway expedites innovation, it may fall short of ensuring clinical robustness and patient safety.

At the same time, the role of AI health startups in collaborating with healthcare providers and driving healthcare innovation has been pivotal. Leading U.S. health systems, such as Mass General Brigham and the Mayo Clinic, have invested millions of dollars in AI and digital health startups^15–17^. Companies like Avenda Health and AEYE Health have received FDA clearance for tools that improve diagnostics^18,19^, while others like Insilico Medicine are accelerating drug discovery with advanced AI systems after raising $255 million in Series C funding^20,21^. Startups have proven instrumental in bridging healthcare gaps, addressing unmet clinical needs, and introducing groundbreaking solutions.

In 2024, AI-focused companies attracted 42% of global digital health funding, marking a historic high and underscoring a shift in investor priorities toward scalable, data-driven health innovations^22,23^. This surge coincides with a global digital health market expected to grow from USD 288.6 billion in 2024 to nearly USD 946 billion by 2030, reflecting a compound annual growth rate (CAGR) of 22.2%^24^. The rapid rise of AI health startups is further supported by a vibrant ecosystem of incubators, accelerators, and strategic partnerships with established healthcare providers, creating fertile ground for innovation^25–27^. Despite significant investment and growing expectations, there is insufficient understanding of how AI health startups have developed across diverse healthcare fields. Specifically, there is a lack of clarity on which healthcare domains have attracted the most venture capital investment and how entrepreneurs and founding teams translate these investments into different types of AI systems that impact healthcare innovations.

Unpacking healthcare domains and entrepreneurial team composition is particularly important in the context of AI-enabled health innovation. Healthcare represents an unusually complex environment characterized by regulatory constraints, specialized knowledge requirements, and strong institutional dependencies. The introduction of AI adds further layers of technological and infrastructural complexity, including advanced technical expertise, data processing capabilities, and integration challenges^28–30^. These combined sources of complexity create structural demands that differ from many other entrepreneurial settings. In particular, they suggest a greater reliance on specialized expertise within founding teams, contrasting with prior theoretical arguments that emphasize the advantages of generalist entrepreneurial profiles^31^. Accordingly, AI healthcare ventures represent a distinct organizational and innovation context, warranting closer theoretical attention compared with traditional models of startup formation and success^32^. Although the transformative potential of AI in healthcare has been widely discussed, research has only recently begun to examine how AI startups navigate the complex terrain of health innovation ecosystems, primarily through systematic review of literature^33–37^. While there is growing interest in the use of AI by established health institutions and large technology companies, early-stage startups—a critical source of innovation—remain underexplored in the empirical literature. Current studies often focus on technical performance, clinical efficacy, or regulatory bottlenecks^33,38,39^ but overlook how startups formulate innovation strategies, pursue partnerships, and engage in market signaling to gain legitimacy and secure funding. Moreover, prior research has tended to focus on sector-level analyses, with limited attention on how institutional and ecosystem-level dynamics—such as alliances with hospitals, research institutions, or health tech platforms—affect startup trajectories^34,36,37,40,41^. Finally, there is a theoretical gap in integrating AI algorithms and health data, health innovation systems, and organizational perspectives to understand how these ventures scale, specialize, or succeed^35,39,40,42,43^.

Our initial analysis shows that AI development trajectories vary substantially across medical domains. Existing studies have examined domain-specific patterns, such as radiology^44^, clinical decision support^45^, mental health, and surgical applications^46^, often emphasizing technological performance and adoption^47^. While this body of work provides valuable insights into AI use within individual specialties, digital health research frequently aggregates findings across medical contexts, implicitly treating healthcare as a homogeneous environment. Such aggregation obscures meaningful differences in knowledge bases, data regimes, regulatory exposure, and clinical workflows across domains. A domain-sensitive perspective is therefore essential for understanding not only technological development but also entrepreneurial dynamics, resource configurations, and investment patterns. Examining AI health startups through the lens of healthcare domain heterogeneity enables a more precise characterization of how ventures navigate distinct institutional and technical conditions, with implications for theory development, entrepreneurial strategy, and venture capital decision-making^43^.

Building on this context, this study seeks to address two related questions: How have startup investments in AI capabilities been distributed across medical domains and types of healthcare innovation? To what extent do founders’ experience and team composition influence the development of AI health startups? Drawing on a large-scale dataset and applying a five-tier AI systems complexity framework, we offer insights into the economics, founding structures, and professional compositions of AI startups that influence AI development in healthcare. Throughout the analysis, we link empirical patterns to the AI systems complexity framework to support a more integrated understanding of how technological characteristics correlate with the emergence of AI health startups and innovation outcomes. While we do not directly assess clinical validation or adoption, our findings provide medical professionals with a clearer view of the innovation landscape and its implications for future care delivery.

## Results

### Geographical and temporal trends in AI health startup formation

**Fig. 1:**
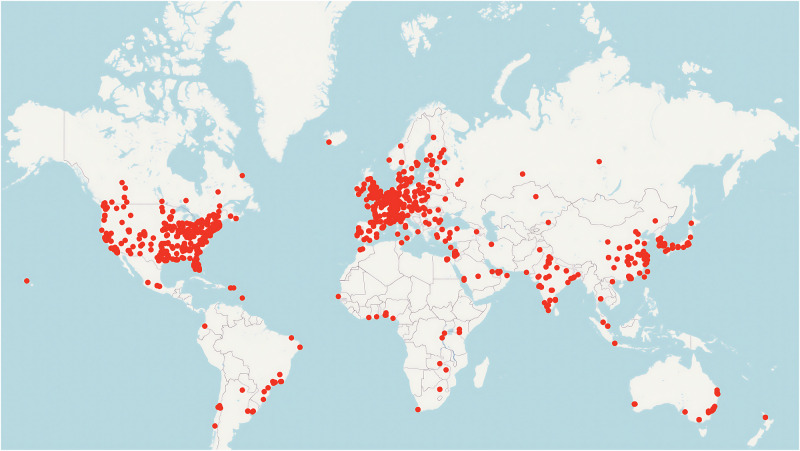
Global geographic distribution of AI health startups. World map showing the location of 3807 AI health startups founded between 2010 and 2024, with each red dot representing one or more startups in that location. Dense clustering is evident in North America, particularly in the United States, around Silicon Valley, Boston, and New York metropolitan areas. European clusters are concentrated in the United Kingdom, Germany, France, and Switzerland. Asia shows significant activity in China, India, Israel, South Korea, and Japan. Notable gaps exist in Africa, South America, and Central Asia, highlighting geographic disparities in AI health innovation. Australia and New Zealand demonstrate a moderate startup presence. The concentration patterns reflect proximity to venture capital, research institutions, and established healthcare innovation ecosystems.

The evolution of AI health startups illustrates a dramatic rise in innovation and activity, followed by a notable decline in recent years. Geographic analysis of AI health startups further underscores disparities in activity and resource distribution. The United States (U.S.) leads the sector with 1609 startups and over $38 billion in funding, followed by China, which shows considerable activity and investment. The map in Fig. 1 reveals a high concentration of startups in North America, Europe, and parts of Asia, particularly in urban and economically advanced clusters. These clusters benefit from proximity to venture capital, specialized talent, and supporting industries, creating favorable conditions for startup growth. Conversely, the scarcity of startups in underrepresented regions, such as Africa and South America, points to untapped potential that could be unlocked through targeted investments and ecosystem-building efforts, including regulatory support, access to capital, and digital infrastructure. Our geographic analysis of AI health startup formation parallels the findings reported in the Global AI Index (https://www.tortoisemedia.com/data/global-ai#rankings), which highlights the concentration of AI capabilities in a few dominant countries, most notably the United States and China, where robust ecosystems, strategic investment, and innovation infrastructure continue to drive AI leadership.

The maps also highlight the role of economic clusters in supporting AI health startups. In the U.S., for instance, dense clusters around Silicon Valley, Boston, and New York emerged as hubs of innovation, underscoring the advantages of geographical proximity to venture capitalists, research institutions, and specialized industries. Similarly, European clusters around London, Berlin, Paris, and Zurich reveal a strong focus on healthcare innovation. These innovation hubs provide access to essential resources, enabling startups to thrive in highly competitive markets. The lack of similar clustering in developing regions, however, poses challenges for local startups, which may struggle to access the resources and networks necessary for sustained growth and scalability.

**Fig. 2:**
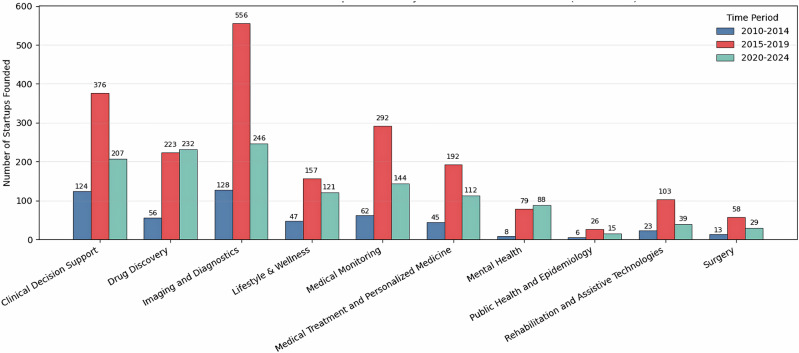
Number of AI health startups founded in the medical domain, 2010–2024. Bar chart showing the number of startups founded across ten medical domains during three time periods: 2010–2014 (blue), 2015–2019 (red), and 2020–2024 (teal). Imaging and Diagnostics experienced the highest growth, with 556 startups founded during 2015–2019, followed by Clinical Decision Support (376 startups) and Medical Monitoring (292 startups). A substantial decline in new startup formation is evident across all domains during 2020–2024, except for Imaging and Diagnostics and Drug Discovery, which maintained moderate activity. Emerging domains, including Mental Health, Public Health and Epidemiology, and Rehabilitation and Assistive Technologies, show limited but increasing activity over time. Numbers above bars indicate startup count per period.

Table 1 shows the number of AI health startups for each medical domain. Figure 2 illustrates the number of AI startups founded across three time periods, 2010–2014, 2015–2019, and 2020–2024, segmented by medical domain. This specific segmentation into 2010–2014, 2015–2019, and 2020–2024 reflects distinct phases in the evolution of AI in healthcare. The years 2010–2014 represent the foundational stage, when traditional machine learning, limited clinical datasets, and slow institutional adoption constrained AI adoption in healthcare. The 2015–2019 period marks a significant shift, characterized by the widespread adoption of deep learning, the release of large medical datasets, clearer regulatory pathways, and substantial growth in venture capital investment, all of which enabled more advanced diagnostic and predictive solutions. Finally, the 2020–2024 period corresponds to the modern era of AI in healthcare, shaped by the acceleration of digital health during COVID-19, broader use of telemedicine, increasing reliance on privacy-preserving machine-learning techniques, and the emergence of foundation models with far greater capabilities^48^.

**Table 1:** Number of AI health startups

Medical domain of AI health startups	Total
Clinical decision support	707
Drug discovery	511
Imaging and diagnostics	930
Lifestyle and wellness	325
Medical monitoring	498
Medical treatment and personalized medicine	349
Mental health	175
Public health and epidemiology	47
Rehabilitation and assistive technologies	165
Surgery	100

From 2010 to 2019, the number of startups increased fourfold, from 512 to 2062, driven by substantial investments and widespread optimism about AI’s potential to revolutionize healthcare. This growth period also witnessed significant diversification across medical domains, with Clinical Decision Support, Drug Discovery, Imaging and Diagnostics, and Medical Monitoring taking center stage. Notably, Imaging and Diagnostics emerged as the leading category, emphasizing the growing focus on precision medicine and early disease detection. Emerging categories such as Mental Health, Public Health and Epidemiology, and Rehabilitation and Assistive Technologies have seen increased activity in recent years, indicating a broadening of AI applications in healthcare. However, the sharp decline in startup entry after 2020 suggests market saturation, shifting investor priorities, and broader economic pressures, indicating a pivotal moment for the sector. As of 2024, about 4% (154) of the startups have been acquired, while 8% (319) have ceased operations. Among the startups that exited the industry, those in the surgery segment had the highest exit rate at 32%, followed by 16% in lifestyle and wellness, and 11% in rehabilitation and assistive technologies. All other segments reported exit rates of 9% or lower.

### Investment and validation of AI health innovations

**Fig. 3:**
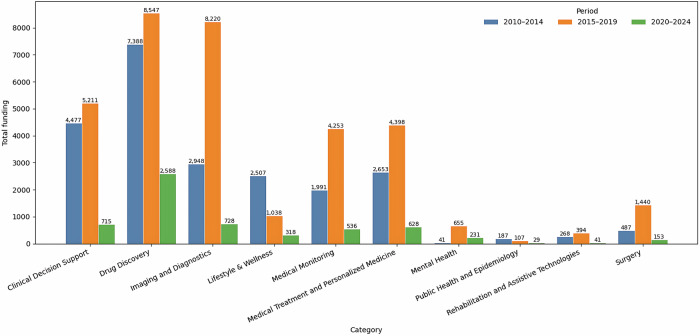
Total funds raised by the medical domain, 2010–2024. This figure presents a bar chart showing total funding (in millions USD) raised by AI health startups across ten medical domains during three time periods: 2010–2014 (blue), 2015–2019 (orange), and 2020–2024 (green). Drug Discovery, Imaging, and Diagnostics attracted the highest total funding, with peak investment during 2015–2019 across most domains. The 2020–2024 period shows a marked decline in funding across all categories, with Imaging and Diagnostics suffering the highest drop. Mental Health, Public Health and Epidemiology, and Rehabilitation and Assistive Technologies received comparatively minimal funding across all periods. Total values are displayed above each bar segment.

To understand the landscape of AI health innovations and the level of complexity associated with each, we analyzed the distribution of startups and their investment funds across varying levels of AI systems complexity. Figure 3 shows that 3807 AI health startups have raised a total of $63.17B since 2010. Most notably, 2062 startups founded from 2015 to 2019 have received $34.26B in total. Based on our detailed analysis across AI systems complexity levels, imaging and diagnostics ($11.87B) and drug discovery ($18.5B) attract the highest levels of funding, largely due to their compatibility with high and advanced complexity AI systems, respectively, especially deep learning in medical imaging and predictive models for pharmaceutical R&D. In contrast, moderate-complexity AI applications such as those supporting clinical decision support ($10.4B) and medical monitoring ($6.78B) are more widely adopted, suggesting that assistive tools designed to augment rather than replace human decision-making more easily integrate into clinical workflows and overcome fewer regulatory hurdles. Finally, the distribution of private equity funding remains uneven across AI complexity levels and medical domains. Lifestyle and wellness ($3.8B) appear to benefit from Assistive AI focused on personalization and guidance, while mental health, public health, epidemiology, and rehabilitation remain significantly underfunded. Additionally, only 38 startups have gone public, primarily in clinical decision support, drug discovery, imaging and diagnostics, and medical treatment, with 7–9 listings per category. The overall investment trend reveals a persistent gap between social health priorities and private investment flows, suggesting targeted funding and policy interventions may be needed to ensure more equitable AI development in healthcare.

**Fig. 4:**
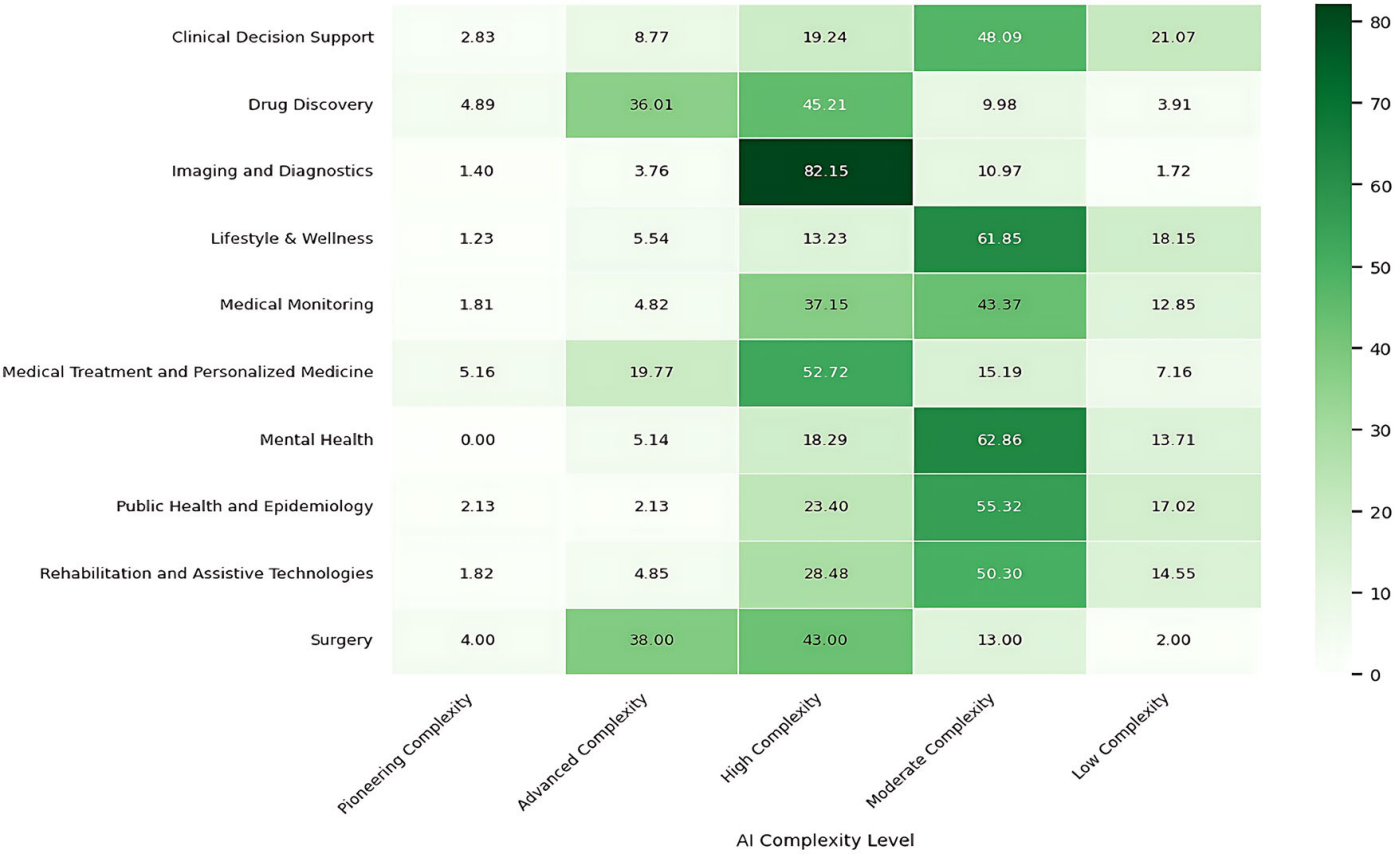
Distribution of AI health startups by medical domain and AI complexity level. Heatmap showing the percentage of startups (n = 3807) operating at each AI complexity level across ten medical domains. Color intensity represents the proportion of startups, with darker green indicating higher concentration. Imaging and Diagnostics shows the highest concentration in High Complexity (82.15%), followed by Medical Treatment and Personalized Medicine (52.72%), reflecting the prevalence of deep learning for medical images and unstructured data analysis. Drug Discovery and Surgery demonstrate substantial representation in Advanced Complexity (36.01% and 38.00%, respectively), indicating adoption of multi-modal AI systems. Lifestyle & Wellness and Mental Health predominantly operate at Moderate Complexity (61.85%, 62.86%, respectively), suggesting a focus on assistive AI tools that augment clinical workflows with human intervention. Clinical Decision Support, Medical Monitoring, Public Health and Epidemiology, and Rehabilitation and Assistive Technologies show balanced distribution across Moderate to High Complexity. Pioneering Complexity remains minimal across all domains (<6%), reflecting technical and regulatory barriers to the development of autonomous-agentic AI systems in healthcare.

Figure 4 shows the proportion of startups operating in each domain across the AI complexity spectrum, from low to pioneering. The map reveals that high and moderate AI system complexity dominate most categories. A small proportion of startups in drug discovery, medical treatment, personalized medicine, and surgery are increasingly shifting towards advanced, pioneering complexity, indicating the need for robust AI knowledge and infrastructure. This is unsurprising given the demands of multimodal and continuous learning systems for integrating diverse datasets, such as genomic and biochemical data, and for operating with minimal human intervention. Next, imaging and diagnostics demonstrates a significant association with high complexity, reflecting the advanced capabilities required to analyze unstructured data such as medical images^12^. These insights underscore the technological and resource-intensive barriers to entry in the health sector, emphasizing the importance of innovation and expertise for startups aiming to succeed in these AI-driven medical domains. Six other domains, including clinical decision support, lifestyle and wellness, and medical monitoring, lean towards moderate to high complexity, reflecting their reliance on structured data and machine learning techniques for practical applications. The map and industry data reveal a nuanced landscape: startups are predominantly deploying moderately to highly complex AI in core technical domains, while deployments in consumer and population health are more cautious. Operational and infrastructure use cases are increasingly validated in practice, though broader adoption hinges on robust governance and strategic ecosystem partnerships^26^.

While the recent decline in startup activity might indicate emerging challenges, regulatory developments provide a contrasting narrative of progress. Since 1995, the U.S. Food and Drug Administration^49^, has approved 1,016 AI/ML-enabled medical devices, with 989 of those approvals occurring in the past decade. FDA approvals have increased significantly, particularly in fields such as radiology (766 devices) and cardiovascular care (103 devices). Consistent with the landscape of AI health startups shown in Fig. 4, the advancement of FDA-approved medical devices reflects the maturation of AI technologies at moderate and high complexity levels and their alignment with stringent regulatory standards, which are critical for widespread adoption. This pattern is evident in the distribution of devices across AI systems complexity: the vast majority fall into Assistive AI and Perceptual AI, as shown in Table 2, which together account for over 97% of all approvals. These complexity levels represent systems that support or augment clinical tasks using structured or unstructured data, such as imaging, and align with well-established clinical workflows.

**Table 2:** AI/ML-enabled medical devices approved by the FDA as of March 2025

AI systems of FDA-approved devices	Total
Deterministic AI	10
Assistive AI	237
Perceptual AI	755
Integrative AI	7
Autonomous-Agentic AI	7

In contrast, more advanced systems, such as Integrative AI and Autonomous-Agentic AI, remain rare, each representing less than 1% of approvals. Their limited presence underscores both the technical and regulatory challenges associated with real-time adaptability and multi-modal data integration^50^. In Table 3, among the top 10 companies with the most devices receiving FDA approvals, six are AI health startups founded between 2011 and 2016. Overall, the FDA approval landscape mirrors startup activity, emphasizing mature, targeted AI applications while highlighting cautious progression toward more complex and autonomous systems.

**Table 3:** Top 10 companies receiving approvals from the FDA as of March 2025

Top 10 companies with FDA approvals	Total
GE Healthcare	71
Siemens Medical Solutions USA, Inc.	46
Canon Medical Systems Corporation	31
Aidoc Medical, Ltd.	25
Shanghai United Imaging Healthcare Co., Ltd.	14
Clarius Mobile Health Corp.	9
Hyperfine, Inc.	9
Viz.ai, Inc.	9
Samsung Medison Co., Ltd.	7
Zebra Medical Vision Ltd.	7

Table 4 maps detailed investment patterns across healthcare application areas and AI systems complexity levels, revealing a distinctly non-linear and context-specific relationship between technological sophistication and funding. Our analysis indicates only a weak positive correlation between overall AI complexity and total funding, suggesting that higher technical sophistication alone does not systematically translate into greater investment. Instead, funding patterns appear to be shaped by the interaction between complexity and domain-specific characteristics, including data structure, regulatory exposure, clinical adoption barriers, and commercialization readiness^41^. In some cases, increasingly complex AI systems coincide with greater capital inflows, while in others, investment peaks at moderate complexity and declines as systems become more technically advanced or autonomous. These findings emphasize that AI capability should be interpreted alongside the practical and institutional realities of each healthcare subdomain.

**Table 4:**
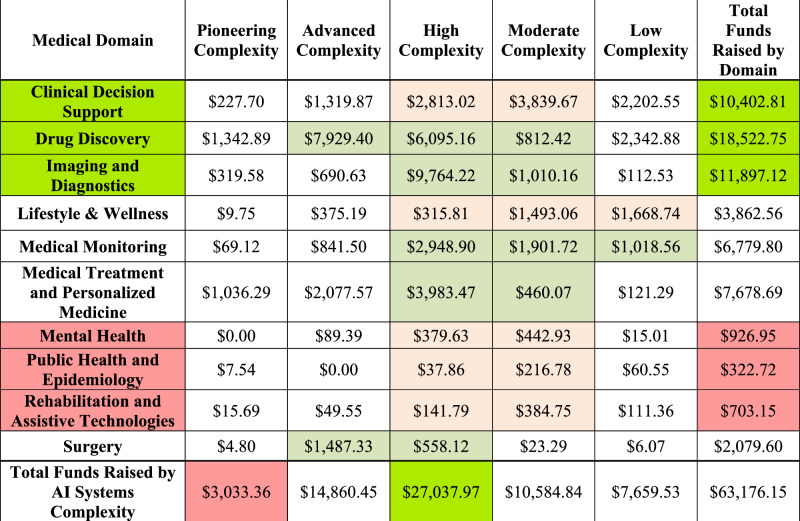
Total funds raised (in millions USD) by medical domain and AI complexity level

Based on Table 4, the concentration of investment and innovation in imaging and diagnostics startups (the second-highest total funding raised after drug discovery) can be better understood through the lens of moderate- to high-complexity AI systems. These domains often leverage convolutional neural networks and other deep learning architectures that are well-suited to standardized, high-resolution imaging data and benefit from relatively mature regulatory pathways in radiology. This alignment between technical capability, data structure, and regulatory clarity lowers barriers to clinical integration and supports investor confidence. In contrast, domains that demand more advanced system integration or real-time decision-making, such as personalized medicine or surgical robotics (the second- and third-highest funding raised at the advanced complexity level after drug discovery), often require more complex AI architectures and higher capital intensity. These higher-complexity tiers not only entail greater development risks but also create entry barriers for clinician-founded ventures that may lack advanced computational expertise. As a result, these areas tend to see more frequent partnerships with technical co-founders or institutional collaborators, particularly hospitals and clinical research centers, who help bridge the validation and implementation gaps. These patterns illustrate how AI system complexity interacts with data availability, regulatory maturity, and human capital to shape innovation trajectories across different healthcare domains.

### The pivotal role of founder experience and team composition

In addition to the challenges of securing investment and navigating technological and regulatory barriers, access to human capital with relevant, diverse experience, particularly at the founding level, is a strategic resource for AI health startups. Founders play a pivotal role in early-stage decision-making, network formation, and shaping the company’s overall direction. Teams with complementary expertise are better positioned to adapt to uncertainty, secure investor confidence, attract partnerships with healthcare institutions, and incorporate ethical, clinical, and technical considerations into product development and deployment^51,52^.

**Fig. 5:**
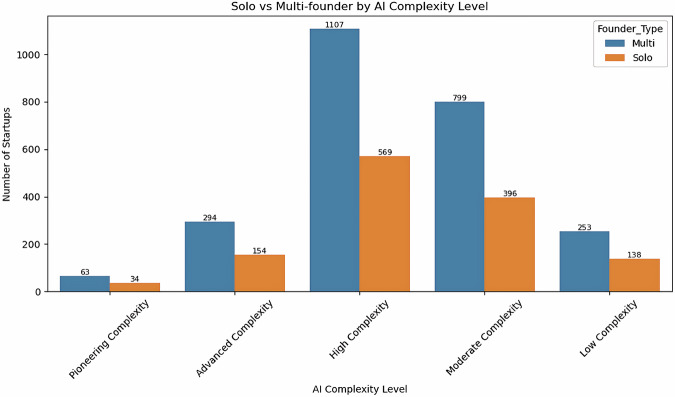
Number of team-based and solo startups by AI complexity. Bar chart comparing the number of solo-founded (orange) and multi-founder (blue) startups across five AI complexity levels. Multi-founder teams consistently outnumber solo founders across all complexity tiers, representing approximately 66% of all startups (2516 multi-founder vs. 1291 solo-founder ventures). The pattern remains remarkably consistent across complexity levels: High Complexity shows the most enormous absolute numbers (1107 multi-founder, 569 solo), followed by Moderate Complexity (799 multi-founder, 396 solo). Advanced Complexity startups show a 2:1 ratio (294 multi-founder, 154 solo), while Pioneering Complexity demonstrates the smallest cohort (63 multi-founder, 34 solo). This consistent preference for team-based founding across all levels of AI complexity suggests that collaborative entrepreneurship is fundamental to AI health ventures, regardless of technological sophistication. Numbers above bars indicate the startup count for each category.

Figure 5 reveals important insights into the founding structures of AI healthcare startups. Of the 3807 startups, 2516 (66.1%) were established by multi-founder teams, while 1291 (33.9%) were initiated by solo founders. Notably, this preference for team-based founding is consistently observed across all levels of AI complexity, with approximately 65% of startups at each tier launched by founding teams. This distribution reflects a strong tendency toward collaborative entrepreneurship in this sector, echoing findings from the broader literature on high-tech and healthcare startups^53–55^. Such a team-based approach is often necessary to address the interdisciplinary challenges of human-AI integration in healthcare^56^. Effectively navigating these challenges requires the convergence of diverse forms of expertise, including medicine, data science, software engineering, and business management. At the same time, teams composed of such varied backgrounds may also be vulnerable to interpersonal conflict, which can hinder collaboration and compromise decision-making.

The formation of founding teams is a strategic response to the multifaceted demands of launching an AI health venture, which often operates within highly regulated, technically sophisticated, and clinically sensitive environments. By combining clinical, technical, and managerial perspectives from the outset, team-based startups are more likely to align their innovations with market needs and regulatory requirements. Additionally, teams with diverse experiences can benefit from access to broader professional networks, more funding opportunities, and a greater capacity for iterative problem-solving. Founding teams also confer distinct advantages in terms of strategic direction, resource acquisition, and organizational resilience. Collectively, these factors—complementary expertise, diverse professional experience, and strategic and organizational strengths—contribute to the scalability and sustainability of AI health startups, positioning founding teams as a critical asset in the venture creation process.

**Fig. 6:**
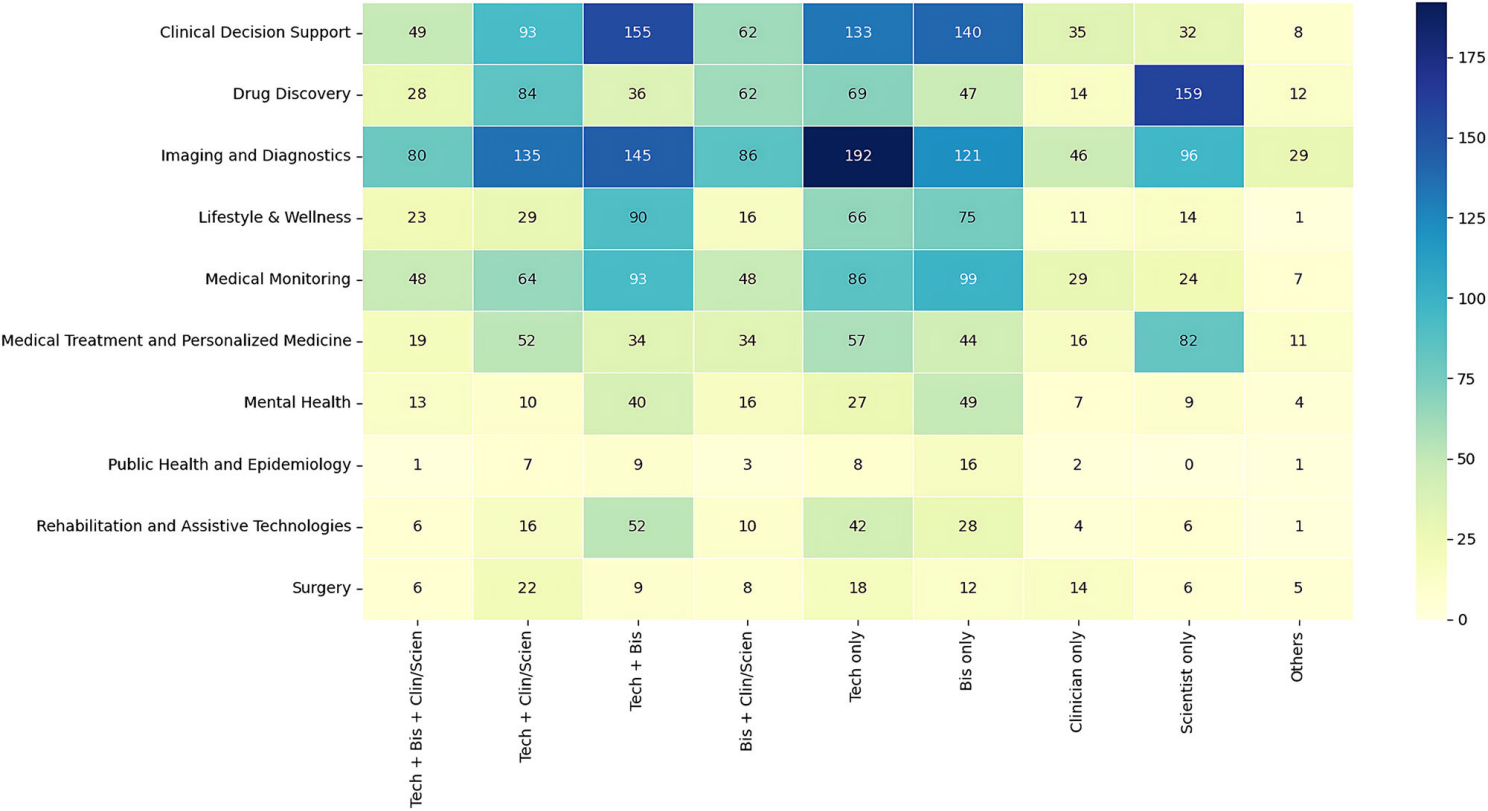
Professional composition of AI health startups by medical domain. Heatmap showing the distribution of 3,807 AI health startups by founders’ professional backgrounds and medical domain. Color intensity indicates startup count. Technical and business expertise dominate across most domains, with technical-cum-business combinations being the most common. Scientist-only founders focus on Drug Discovery, Medical Treatment, and Personalized Medicine. Clinical practitioners remain underrepresented in founding roles across all medical domains, with business and technical founders significantly outnumbering clinicians in leadership positions.

**Fig. 7:**
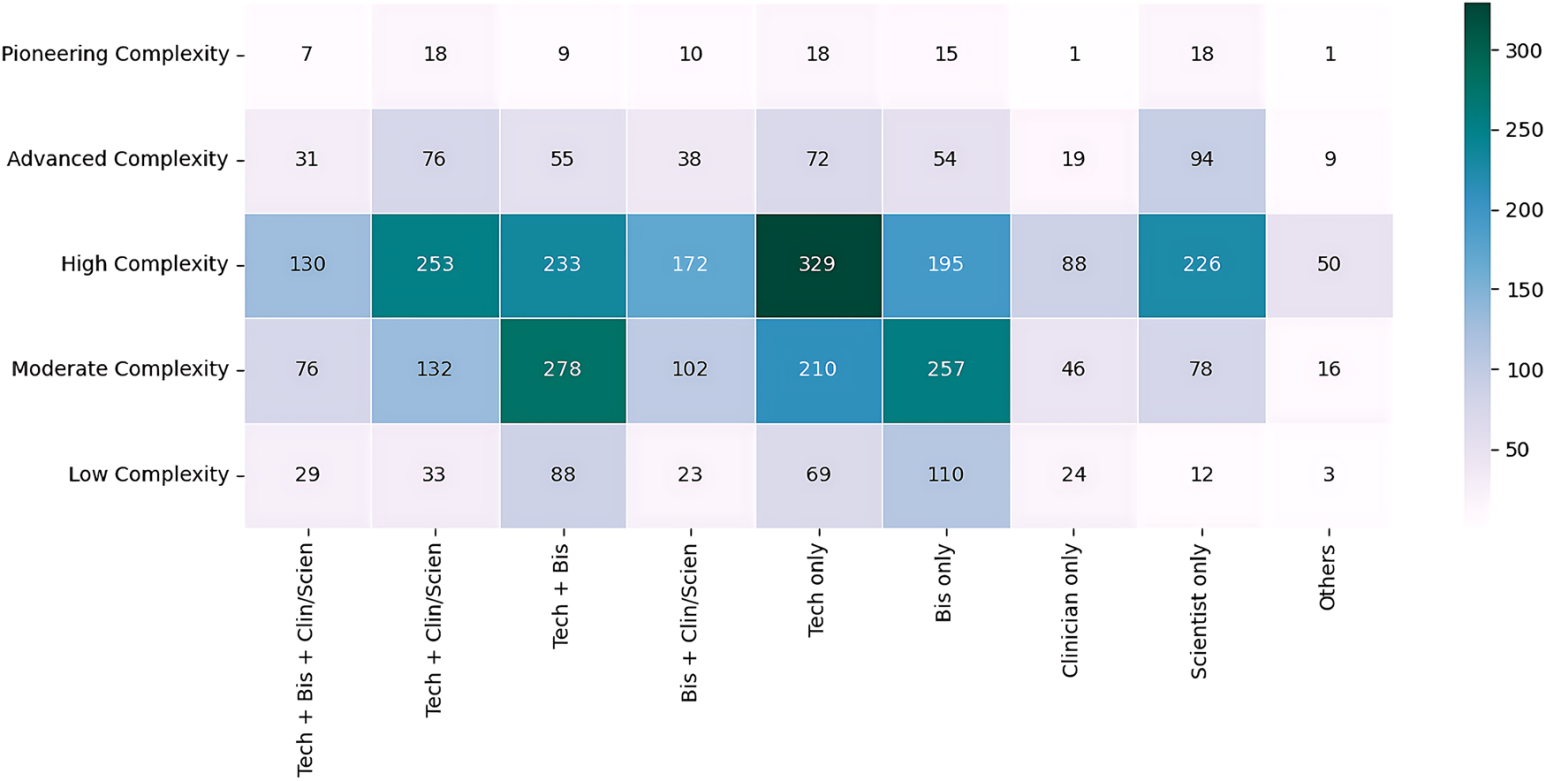
Professional composition of AI health startups by AI complexity. Heatmap showing the distribution of 3807 AI health startups by founder professional background and AI complexity level. Color intensity indicates startup count. High-Complexity startups are predominantly founded by technical founders, particularly technical-only founders or those with technical backgrounds combined with business or scientific and clinical expertise. Moderate Complexity ventures show the highest concentration of founders with technical-business and business-only backgrounds. Startups at the Advanced and Low Complexity levels exhibit a broad range of expertise, whereas clinical practitioners remain underrepresented across all levels of complexity.

Figures 6 and 7 present the professional composition of founding teams and solo founders from 3807 startups across medical domains and AI complexity levels. The data reflects not just the experts in the founding teams but the dominant professional backgrounds shaping each startup’s foundation. In many cases, teams are composed entirely of individuals with the same professional background, while others span technical, business, clinical, and scientific expertise.

Our descriptive analysis shows that most AI health startups are co-founded by two or more individuals, with an average team size of 2.14 (SD = 1.15). In Fig. 6, technical expertise is the most common foundation for startup formation, either combined with business expertise, as seen in 663 (17%) startups, or as the sole domain of expertise in 698 (18%) startups, out of which 410 (11%) are solo-founder startups and 288 (8%) are team-based startups. This pattern underscores the central role of technical capability in shaping the early direction of AI health ventures, especially involving highly complex AI systems in medical domains such as clinical decision support, imaging, and diagnostics. Business expertise is the next most common professional background among founders. Of the 631 (16.6%) startups led by business-only founders, 454 (12%) are solo-founder startups, while only 177 (4.6%) are team-based startups.

In contrast, clinical experts, particularly founders with direct patient-care experience, play a more limited role. In Fig. 6, 178 startups were founded by clinicians only, while 135 of these were by solo clinician-founders, less than 5% of the total sample. Although clinical insight is essential for healthcare innovation, founding teams tend to rely more heavily on medical scientists with academic or research backgrounds. Specifically, scientist-only teams accounted for 161 (4.2%) startups, while solo scientist-founders accounted for 267 (7%).

Figure 7 shows that AI health startups involved in high-complexity AI systems are predominantly founded by technical-only (329) or technical with business (233) or scientific and clinical (253) expertise. Moderate-complexity ventures show the highest concentration of founders with a technical-business background (278) and a business-only background (257). Clinical practitioners remain underrepresented across all complexity levels, while advanced and low complexity startups demonstrate diverse expertise that is relatively well represented.

The two figures (Figs. 6 and 7) indicate that AI health startups generally include technical and scientific expertise, with business skills commonly present and clinical expertise less frequently or selectively involved in team composition. This pattern may be influenced by factors such as limited entrepreneurial training for clinicians, time constraints, or regulatory requirements. It also highlights the growing importance of scientific research and data science in developing AI-based healthcare solutions. These findings are consistent with survey results^57^, which show that a higher proportion of researchers (37% of 2284 respondents) than clinicians (26% of 1007 respondents) have used AI tools for work-related purposes.

Moreover, the underrepresentation of clinician-exclusive or clinician-dominant teams may affect the alignment of these AI solutions with real-world clinical needs and workflows. While scientists and technologists can provide the foundational tools for innovation, the absence of front-line healthcare practitioners in leadership roles may lead to a disconnect between problem identification, technical solutions, and patient care outcomes. To assess whether the low representation of clinicians simply reflects investor selection (i.e., investors favoring teams with stronger technical/business profiles), we conducted a split-sample analysis comparing funded and non-funded AI health startups. If investor screening were the primary driver, we would expect clinician under-representation to be concentrated among funded firms; instead, the observed patterns remain qualitatively stable, with clinically involved teams present at all funding levels rather than systematically excluded. Thus, our data not only highlight current trends in founding team composition but also raise important questions about how entrepreneurial ecosystems can better support clinician involvement to ensure the clinical relevance and adoption of AI healthcare technologies.

### Gendered patterns in founding structures and expertise across AI health startups

Table 5 provides a gender representation among founders of AI health startups. Overall, male founders overwhelmingly dominate the landscape, comprising 6872 individuals (84%), compared to 1293 female founders (15.8%). Although the proportion of women remains low, the 15.8% figure falls within the 10–15% range reported in comparable datasets of startups founded from 2010 onwards^58–61^. In Dowd’s^58^ report, women comprised just 13.2% of startup founders in 2023, which was the lowest since 2018. Among industry sectors, healthtech had the highest percentage of female founders.

**Table 5:** Gender difference by founder background

Founder background	Female	Male	% Female
**Business**	447	2017	18.1%
**Clinical**	154	760	16.8%
**Scientific**	351	1324	20.1%
**Technical**	332	2748	10.7%
**Others**	9	23	28.1%
**Total**	1293	6872	15.8%

Further disaggregation by founder expertise reveals meaningful variation in gender representation across domains. Women are most represented among scientific founders, comprising 20.1%, reflecting the growing participation of women with advanced degrees, particularly PhDs in biomedical and life sciences. Business and clinical founders follow with 18.1% and 16.8% female representation, respectively. Strikingly, technical founders exhibit the lowest female participation rate at 10.7%, despite being the largest group driving AI health ventures.

Nevertheless, the continued underrepresentation of expertise among female founders, particularly in technical founding roles, calls for proactive, ecosystem-level interventions, such as mentorship programs, funding opportunities, and institutional support structures, to sustain and amplify female participation in shaping the future of AI in healthcare. These disparities also raise important considerations for building more diverse founding teams capable of developing equitable and context-sensitive healthcare technologies^51,55^.

**Fig. 8:**
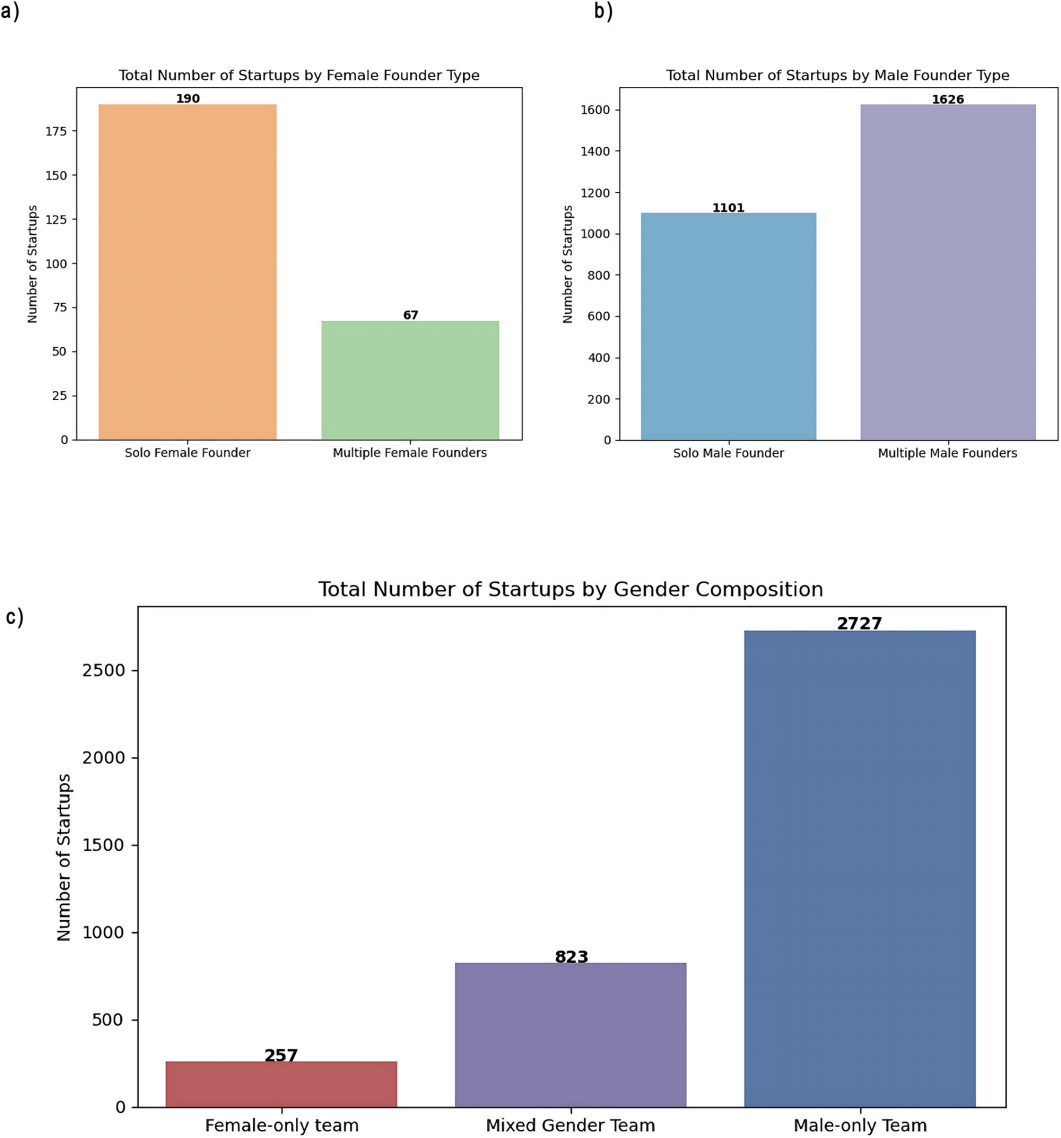
Distribution of AI health startups by gender. **a** Distribution of startups by gender composition showing male-only teams comprise the majority (2727 startups, 71.6%), followed by mixed-gender teams (823 startups, 21.6%), and female-only teams (257 startups, 6.75%). **b** Female-only startups by founding structure reveal a strong skew toward solo entrepreneurship, with 190 solo female founders (74%) compared to only 67 multi-female founder teams (26%). **c** Male-only startups demonstrate more balanced founding structures, with 1626 multi-male founder teams (60%) and 1101 solo male founders (40%). The differences in founding patterns between female-only and male-only ventures highlight the distinct challenges men and women face in AI health entrepreneurship.

Further analysis of gender composition across AI health startups in Fig. 8a–c reveals a pronounced skew toward male-only founding teams and solo male founders, who together account for approximately 71.6% of all startups in the sample. In contrast, mixed-gender founding teams represent about 21.6%, while startups founded exclusively by women comprise just 6.75% of the total, highlighting the persistent gender imbalance in the entrepreneurial landscape.

When disaggregating by founding structure, the pattern is especially notable among 257 female-only startups: 74% of exclusively female-founded ventures are led by solo founders, compared to just 26% involving multiple female founders. This contrasts with 2727 male-only startups, where founding structures are more balanced: 40% are led by solo male founders, and male teams co-found 60%. These trends suggest that women in AI health entrepreneurship are not only underrepresented but also more likely to pursue solo ventures, potentially reflecting broader structural barriers to team formation, resource access, or professional networks.

### Domain-specific gender patterns (Fig. 9a, b)

**Fig. 9:**
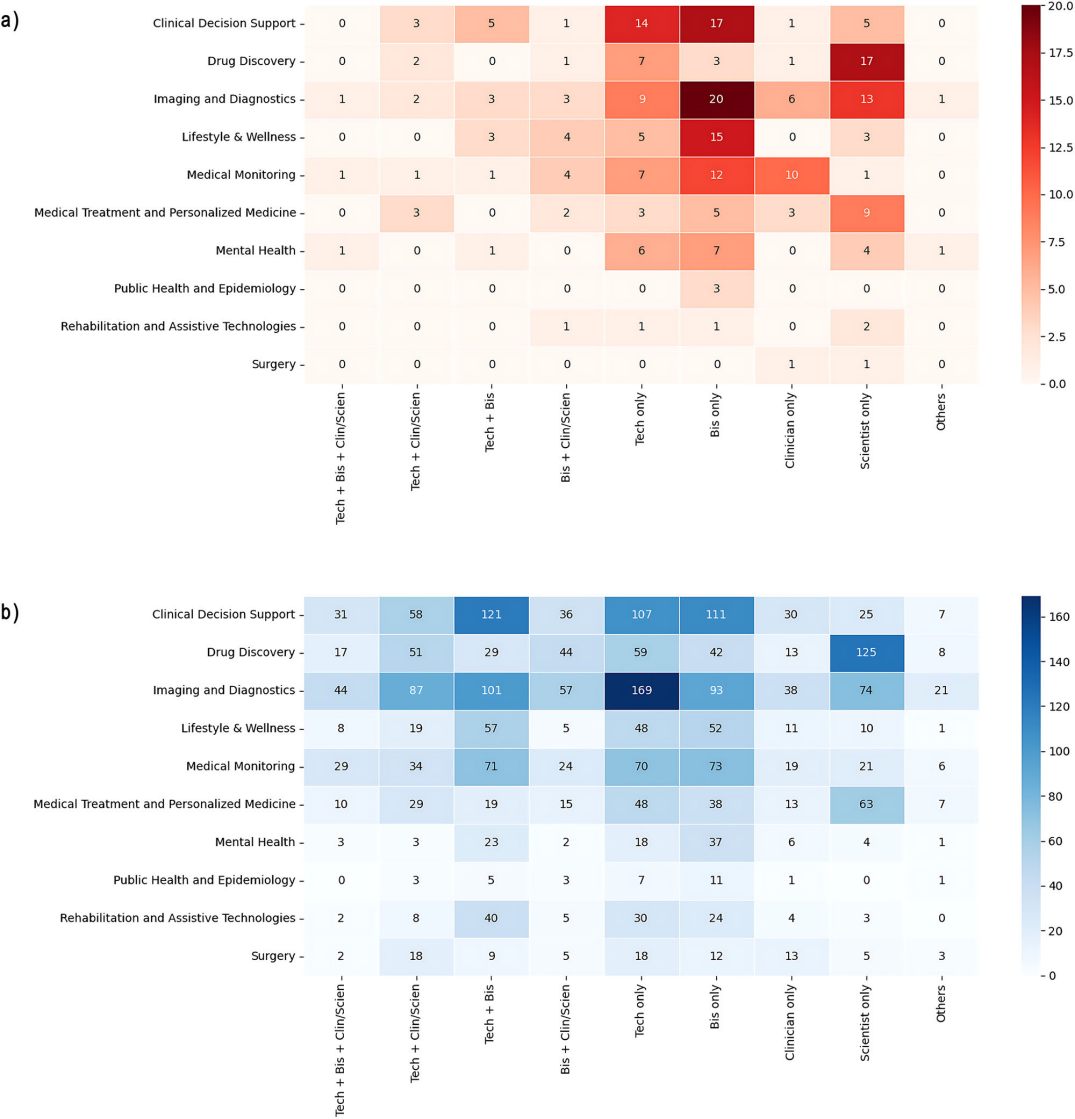
Professional composition of startups by gender and medical domain. **a** Female-only startups (n = 257) show concentration in Clinical Decision Support and Imaging and Diagnostics, with business-only founders (dark red cells) being most prevalent. Scientist-only founders appear prominently in Drug Discovery and Medical Treatment. Clinical-only founding teams are notably sparse, with Medical Monitoring showing the highest count at 10 founders. **b** Male-only startups (n = 2727) demonstrate substantially higher absolute numbers across all domains and founder types. Technical-only founders (dark blue cells) dominate in Imaging and Diagnostics (169) while scientist-only founders concentrate in Drug Discovery (125). Technical-business combinations are most common in Clinical Decision Support (121) and Imaging and Diagnostics (101). The comparison reveals that female-only startups are more focused on business-led ventures. In contrast, male-only startups exhibit stronger representation of technical backgrounds, suggesting different experiential pathways into AI health entrepreneurship.

Figure 9a, b presents the distribution of female-only and male-only AI health startups across medical domains, combining both solo and team-based founders. A strong gendered concentration emerges, with both female- and male-only startups being heavily represented in Clinical Decision Support and Imaging and Diagnostics. However, female-only startups are more frequently led by founders with business-only backgrounds, while technical founders predominantly lead male-only startups. This divergence in founders’ expertise suggests gender differences in pathways into AI healthcare innovation. This is consistent with broader research highlighting gendered differences in technical versus managerial entry into entrepreneurial ecosystems^53,54^.

### Founding structures—female startups (Figs. 10a and 11a)

**Fig. 10:**
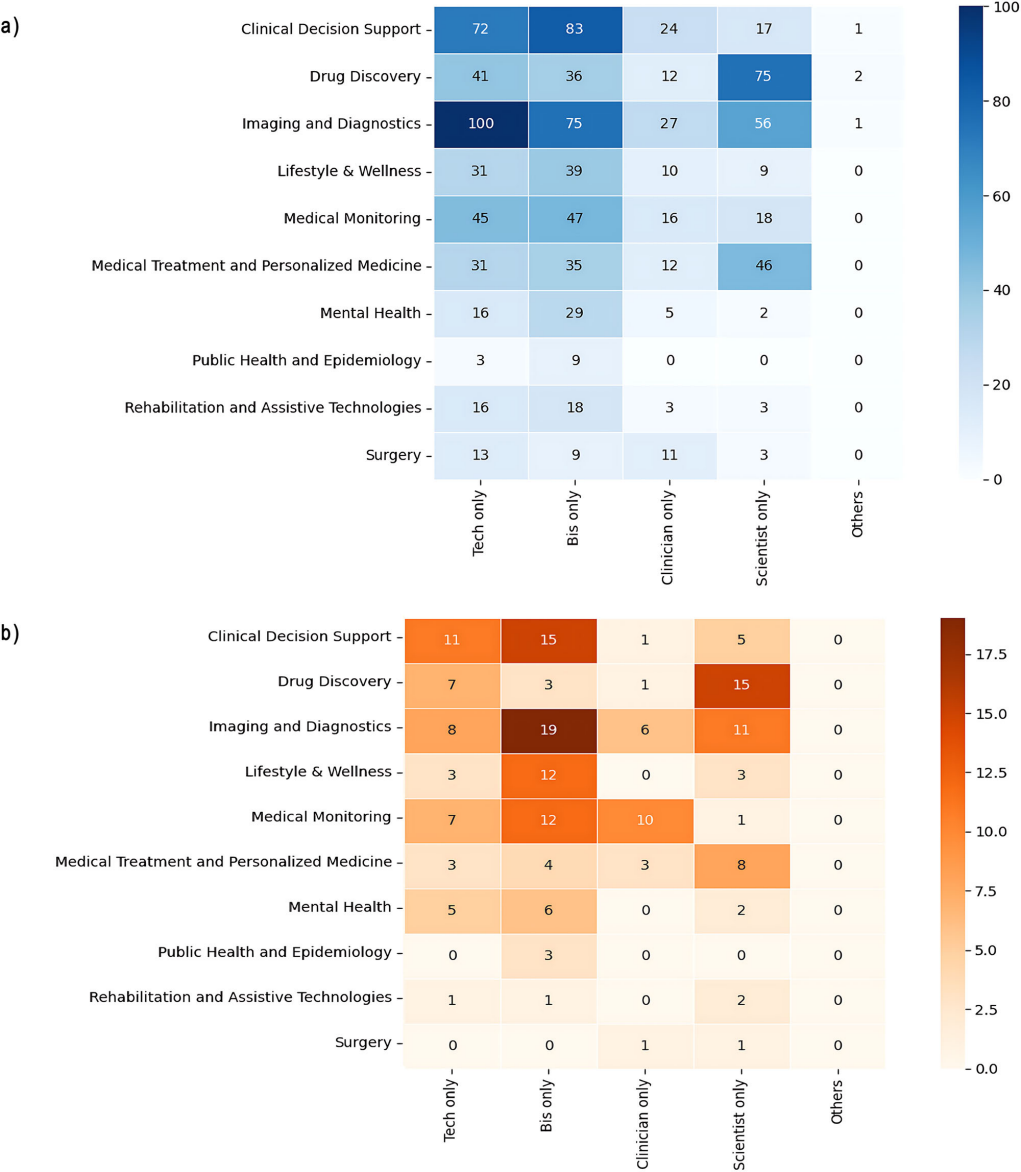
Professional backgrounds of solo-founders by gender. **a** Solo female founders (n = 190) predominantly have business backgrounds, particularly in Imaging and Diagnostics (19) and Clinical Decision Support (15). Scientist-only female founders concentrate in Drug Discovery (15). Clinician-only female founders remain sparse, with Medical Monitoring showing the highest representation (10). Female solo founders with clinical-only backgrounds are present in only six medical domains. **b** Solo male founders (n = 1101) show stronger technical representation, with technical-only founders most prominent in Imaging and Diagnostics (100) and Clinical Decision Support (72). Business-only male founders are also most represented in Clinical Decision Support (83) and Imaging and Diagnostics (75). Scientist-only male founders concentrate in Drug Discovery (75) and Imaging and Diagnostics (56). Male solo founders with clinical-only backgrounds are underrepresented across domains, except in Surgery. The comparison reveals gendered patterns in solo entrepreneurship: female solo founders are more likely to have business backgrounds, while male solo founders are more likely to have technical expertise.

**Fig. 11:**
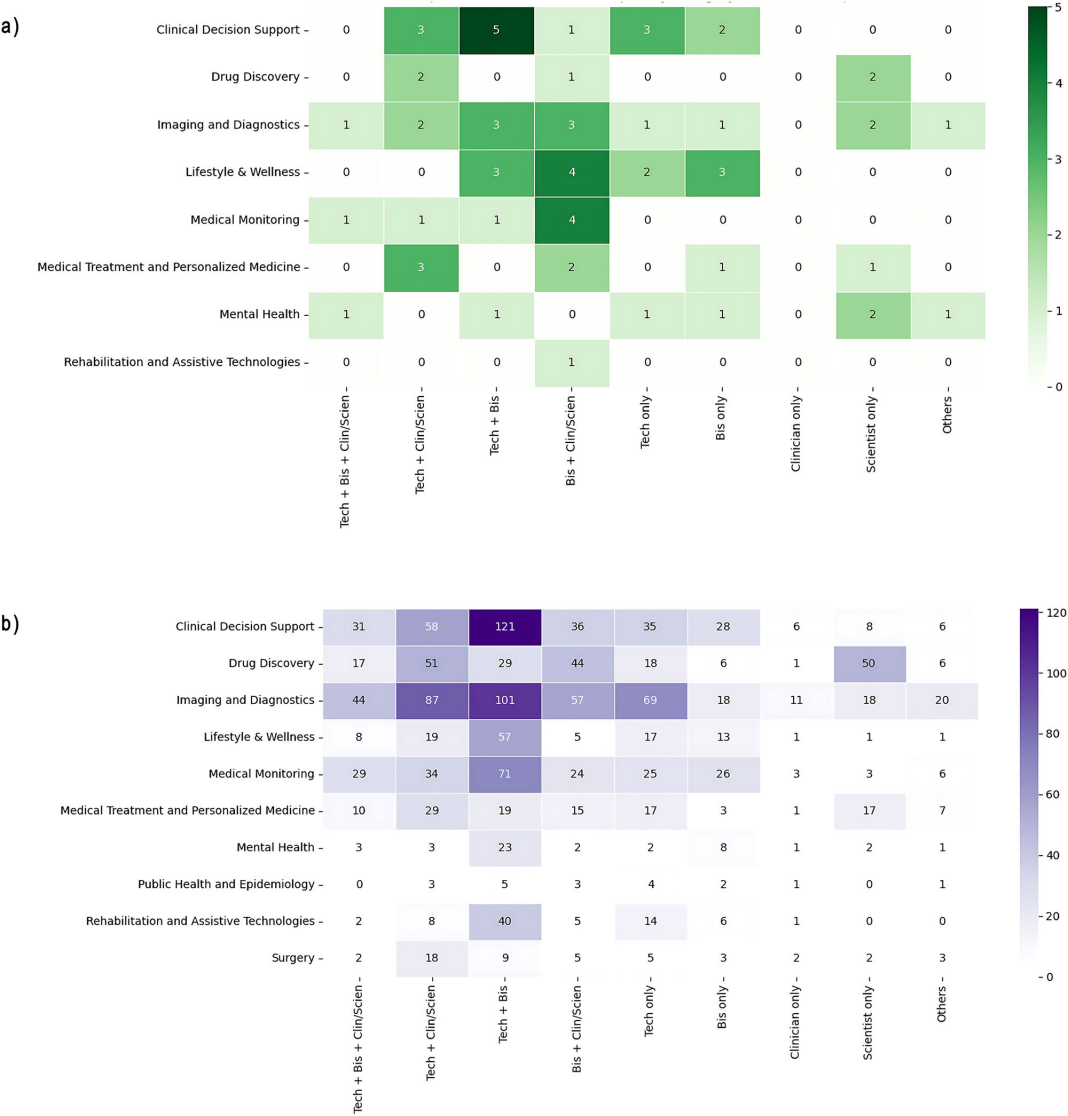
Professional backgrounds of team-based founders by gender. **a** Female-only teams (n = 67) show limited formation across domains, with the highest representation in Clinical Decision Support (14) and Imaging and Diagnostics (14). Business-clinical combinations (dark green) are the most common in Lifestyle & Wellness (4) and Medical Monitoring (4), while Technical-business teams lead in Clinical Decision Support (5). Notably absent are female-only teams in Public Health, Epidemiology, and Surgery. **b** Male-only teams (n = 1626) demonstrate substantially higher numbers across all domains and team compositions. Technical-business combinations (dark purple) dominate in Clinical Decision Support (121), Imaging and Diagnostics (101), and Medical Monitoring (71). Technical-scientific/clinical teams are well represented in Imaging and Diagnostics (87), Clinical Decision Support (58), and Drug Discovery (51). The stark difference in team formation patterns—with female-only teams representing just 4% of all team-based startups—suggests significant challenges women likely experience in forming all-female founding teams in AI health entrepreneurship.

Figures 10a and 11a show that 190 startups are led by solo female founders, compared to 67 founded by female-only teams, highlighting a strong individual entrepreneurial presence among women. This solo dominance persists across medical domains. For instance, in Imaging and Diagnostics, 44 startups are led by solo female founders compared to 14 by all-female teams. Notably, no female-only teams were identified in Public Health and Epidemiology or Surgery (Fig. 11a), indicating limited representation of all-female teams in some specialties.

Among solo female founders, the most prevalent professional background is business, with 75 founders possessing business expertise, followed by 48 founders with a scientific background (Fig. 10a). Nineteen of the solo business founders are concentrated in Imaging and Diagnostics, while only one team startup has a business-only composition. In Drug Discovery, 15 of the 26 solo female founders have a scientific background, compared to just two female-only teams with similar expertise (Figs. 10a and 11a). These findings suggest that women with scientific or business expertise are more likely to launch ventures individually rather than as part of all-female teams. Taken together, the above findings align with prior studies indicating that female entrepreneurs in science-based and high-tech fields often face higher barriers to entrepreneurship due to constraints on forming complementary teams and accessing network resources^62,63^.

### Founding structures—male startups (Figs. 10b and 11b)

Conversely, male-only founder startups exhibit greater balance between solo and team-based structures. In Imaging and Diagnostics, for example, 259 solo male-founded startups are matched by 425 team-based male-only ventures. Across medical fields, most male-only startups have teams with both technical and business expertise (475 teams), followed by technical and scientific/clinical (223), technical-only (206), and business and scientific/clinical expertise (196). Similar to female-only startups, male founders who are clinical or scientific-only are less likely to form startups alone or in teams. This supports prior findings that interdisciplinary team formation is especially critical in health technology startups^55^.

In summary, this comparative analysis underscores a notable difference in team dynamics: exclusively female-founded ventures are skewed toward individual entrepreneurship, whereas male-founded ventures exhibit greater balance between solo and team startup models. Among female-only teams that do form, the most common composition combines business and clinical/scientific expertise (16 teams). Although 22 solo female founders have clinical expertise, no female-only startups have been founded solely by clinicians. This pattern may reflect a structural preference, or necessity, for clinically trained women to pursue ventures individually or within mixed-gender teams, potentially due to limited access to like-minded co-founders or institutional support. The low presence of clinician-only teams may also result from the high demands and time constraints of clinical practice, which make team entrepreneurship more difficult unless complemented by partners with managerial or technical expertise. Together, these patterns point to persistent barriers in forming diverse, interdisciplinary founding teams, an important consideration for efforts to broaden participation and leadership in AI health innovation.

## Discussions

Our findings shed light on the intricate dynamics shaping the AI health startup landscape, moving beyond speculative narratives to offer empirical insights into how these ventures are evolving. By analyzing 3807 AI health startups using a five-tier AI systems complexity framework, we observe significant variability in both technological progress and healthcare innovation. Most startups operate at lower levels of complexity, focusing on assistive or perceptual AI rather than fully integrated or autonomous systems. This reflects not only technological limitations but also the regulatory, clinical, and economic barriers that complex AI systems face in gaining traction.

The analysis highlights dominant trends in innovation focus and capital allocation. Clinical decision support, drug discovery, and imaging and diagnostics remain the most heavily funded and populated domains within the AI-driven health ecosystem. These areas are favored due to their scalability, standardized data formats, and more established clinical integration pathways. While this concentration may reflect pragmatic business strategies, it also raises concerns about potential neglect of less commercially attractive but equally critical areas such as mental health, public health, and rehabilitation medicine.

Geographic distribution also reveals systemic imbalances. While innovation hubs like Silicon Valley and Boston continue to lead in startup formation and investment, startups in emerging regions struggle to access venture capital, mentorship networks, and interoperable digital infrastructure. This pattern is consistent with entrepreneurship research showing that the geography of venture capital shapes which ventures gain traction and their locations^64^, and that performance differentials within geographic clusters reflect uneven access to specialized knowledge, talent, and institutional support^65^. Despite the global discourse on democratizing digital health, the sustained dominance of established U.S. clusters underscores the persistent barriers to broader, more equitable innovation diffusion, leading to a divided health delivery system in which AI-driven solutions depend on proximity to venture capital funding rather than patient needs. This trend contradicts the WHO’s Universal Health Coverage (UHC) principle.

Our findings also highlight persistent structural asymmetries in team composition. Founding teams are predominantly composed of individuals with technical and business backgrounds, while clinical expertise, especially from those with direct patient-care experience, remains underrepresented. The entrepreneurship literature has long established that founding team composition is a key determinant of new venture performance, with meta-analytic evidence showing that the diversity and functional balance of founding teams shape growth trajectories, innovation outcomes, and access to resources^53,54,66^. Research on technology-based ventures further demonstrates that the type of founder human capital—whether technical, managerial, or industry-specific—differentially affects venture growth, with commercially oriented experience proving especially consequential^67^. In the AI healthcare context, however, clinical expertise introduces a distinct and underexplored dimension of founder human capital. This gap is significant, as clinician involvement is critical not only for shaping clinically relevant innovations but also for building trust, ensuring usability, and integrating AI tools into complex end-to-end diagnostic and treatment workflows^68,69^. One pathway to mitigate this gap is through early-stage partnerships between AI startups and healthcare professionals, particularly in co-designing solutions, validating clinical utility, and navigating adoption barriers such as trust, transparency, and workflow integration^70^. These collaborations can address key challenges identified in clinician adoption, such as the need for explainability, clinical validation, and training, and offer more context-sensitive AI applications. Moreover, we observe a notable gender imbalance: female representation is especially low among technical founders, and solo female founders are significantly more likely to lead ventures alone rather than in teams. These structural patterns shape not only innovation trajectories but also the types of problems startups prioritize and the populations they are most likely to serve.

Together, these findings suggest that while AI in healthcare continues to advance rapidly, important gaps remain in domain diversity and equitable access to the innovation ecosystem. Addressing these gaps will require intentional interventions that go beyond technical advancement and extend into education, funding policy, and ecosystem design.

This study provides an empirical portrait of the evolving AI health startup landscape. While our findings are descriptive and do not establish causal effects, they raise several critical questions that future research, especially by interdisciplinary teams in digital medicine, could address using longitudinal or experimental designs.

*For medical professionals and clinical researchers*, our work highlights the underrepresentation of clinical expertise in early-stage AI health ventures. This pattern suggests the need to explore how clinician involvement (or lack thereof) shapes the clinical relevance, ethical framing, and usability of AI-enabled tools. Future research could investigate:

- Does clinician involvement in founding teams or advisory roles lead to more clinically validated or adoptable AI solutions?
- How do different forms of clinical expertise (e.g., primary care vs. specialty care, rural vs. urban settings) influence product design or target populations?
- Do formal partnerships with clinical organizations and research institutions accelerate regulatory approval, reimbursement pathways, or trust from frontline providers?

Future research could ground these questions in theories of professional hybridity and boundary-spanning innovation^71^, which explain how professionals bridging distinct institutional logics, such as medicine and technology, mediate legitimacy, managerial practices, and the implementation of innovation. Clinician-founders and advisors often function as boundary spanners, translating medical practice needs into data science models and regulatory pathways^72^. This line of inquiry aligns with institutional theory^73^, particularly in examining how legitimacy is conferred through medical expertise in entrepreneurial ecosystems. Moreover, theories of co-production in healthcare innovation^74,75^, could inform future research on how collaborative design processes with clinicians affect validation cycles, patient outcomes, and adoption trajectories.

*For innovation scholars and entrepreneurship researchers*, our analysis opens questions about the relationship between founding team composition and technological development. It is essential to understand how early team and venture configurations imprint on venture trajectories, shaping strategic direction, resource acquisition, and the likelihood of securing external investment^53,54,76^. Future studies could explore:

- What combinations of technical, clinical, and entrepreneurial expertise are most conducive to advancing complex AI health innovations?
- To what extent do initial founding team configurations shape a startup’s access to capital, data partnerships, or regulatory pathways?
- How do team configurations mediate access to formal partnerships with healthcare providers, payers, or pharma companies?

These research opportunities can draw more directly from the richness and depth of entrepreneurship literature to advance theories of human-AI collaboration, ethical alignment, and technological framing within the healthcare ecosystem^77–79^. For instanceRaisch and Krakowski^79^, identified the automation–augmentation paradox in AI management, a tension that is especially salient in healthcare, while Murray et al.^78^ theorized forms of conjoined human-technology agency in organizations, offering a lens for studying how clinicians and AI systems jointly produce diagnostic or treatment decisions. The variety of partnership and validation approaches can be further interpreted through frameworks on dynamic capabilities and legitimacy transfer^80,81^. Finally, the diverse commercialization trajectories observed across AI complexity levels resonate with existing work on entrepreneurial experimentation and digital platform evolution^82–85^.

*For policymakers and funding agencies*, our findings underscore persistent geographic and expertise disparities in the AI-driven health sector. Rather than treating AI innovation as a global phenomenon, there is a need for region-specific support that includes capacity building, regulatory guidance, and access to validation infrastructure. Questions that warrant further attention include:

- What interventions (e.g., targeted grants, incubators, regional testing beds) effectively support AI health startups in underserved geographies?
- What governance mechanisms are needed to ensure AI in healthcare is equitable, bias-mitigated, and beneficial across diverse patient populations?
- How can regulatory frameworks evolve to ensure safety, efficacy, and accountability for continuously learning and adaptive AI systems in healthcare?

Our findings indicate persistent gender imbalances in AI health startup founding teams, with women-founded ventures receiving less investment and exhibiting different founding structures than their male counterparts^63^. These patterns are consistent with entrepreneurship research documenting the gender gap in venture funding and innovation performance outcomes^86,87^. Policymakers and funding agencies should therefore incorporate gender-equity objectives into innovation support programs. This may include targeted funding streams for women-led AI health ventures, diversity requirements or incentives in public grant evaluations, and capacity-building initiatives that strengthen opportunities for female founders in technical, clinical, and entrepreneurial roles. Addressing these disparities is essential not only for fairness but also for expanding the diversity of problems addressed, improving market responsiveness, and fostering more inclusive and equitable AI healthcare innovation ecosystems, especially given the persistence of subjective decision-making biases that disadvantage female founders in early-stage investment contexts^88^.

*For healthcare institutions*, the data reveal where innovation is concentrated (e.g., decision support, diagnostics) and where it remains sparse. As AI systems evolve toward greater complexity, integrating multimodal data, autonomous learning, and real-time decision support, healthcare institutions face new challenges in management, validation, and governance. In particular, the following questions merit attention:

- How do healthcare organizations adapt their operational and governance models to manage, validate, and monitor increasingly autonomous AI systems?
- How do evolving human–AI interactions in clinical decision-making influence patient outcomes, professional roles, and accountability structures?
- What organizational capabilities and leadership practices are needed to ensure that the adoption of complex AI systems enhances, rather than fragments, patient-centered care and equity?

AI governance in healthcare remains fragmented, with many existing frameworks assuming extensive resources and offering limited guidance for institutions at earlier stages of readiness^89,90^. Recent efforts to address this include maturity-based models such as the Healthcare AI Governance Readiness Assessment (HAIRA), which provides tiered progression benchmarks across critical governance domains^91^, and empirical case studies demonstrating how practical frameworks like the People, Process, Technology, and Operations (PPTO) model can be adapted to specific institutional contexts to establish formal oversight structures^92^. These contributions underscore that governance is not a one-time design exercise but a continuous, adaptive process. Meanwhile, research on human–AI collaboration in clinical settings reveals that interaction patterns remain largely tool-centric, with the quality of collaboration depending heavily on how and when AI outputs are presented and whether clinicians retain genuine agency to override algorithmic suggestions^93^. This line of inquiry connects to theories of organizational learning and dynamic capabilities in complex adaptive systems^94^, as well as recent frameworks in sociotechnical systems design and AI governance in healthcare^95,96^. Together, these perspectives can help explain how institutions evolve alongside increasingly intelligent technologies and what governance mechanisms ensure safety, trust, and continuous improvement in AI-enabled healthcare.

As AI continues to shape the future of healthcare, the central question remains open: Who will be the great physicians in this new era—the humans, the machines, or the teams they form together? As Lee et al.^13^, cautioned, for the foreseeable future, AI cannot be used in medical settings without direct human supervision due to the risk of diagnostic errors. In addition to concerns about accuracy, ongoing issues such as bias, data quality, privacy, and transparency are often not fully considered by AI companies and investors prioritizing rapid deployment, and may not be thoroughly addressed by clinicians, health authorities, and policymakers facing fragmented oversight^97^. These issues underscore that the path toward AI-enabled medicine is not merely technical, but profoundly ethical and systemic, demanding sustained attention, participatory governance, and critical reflection from all stakeholders.

Beyond the specific contours of AI health entrepreneurship, the patterns we identify, concentrated investment in technologically mature domains, uneven geographic participation, and persistent gaps in team diversity, raise important questions about whether similar dynamics are unfolding in other AI-intensive sectors such as finance, education, and public administration. Future research should explicitly examine whether these trends are mirrored in adjacent domains, and whether the mechanisms driving concentration and exclusion operate in comparable or distinct ways. Such comparative inquiry would open new avenues for understanding how structural conditions, institutional maturity, and market logics shape the development of AI across different societal contexts.

At the same time, the limited diffusion of innovation capacity to under-resourced regions in our study underscores the risk of widening international disparities in access, adoption, and benefit. As countries explore national AI strategies and health systems worldwide experiment with digital transformation, our findings highlight the importance of globally coordinated investments in talent pipelines, clinical collaboration, regulatory capacity, and pluralistic innovation ecosystems. Strengthening these foundations, particularly in lower-resource settings, will be essential for ensuring that the benefits of AI-enabled healthcare become more transferable across regions and adaptable to diverse health-system needs, ultimately supporting more inclusive and equitable pathways to innovation.

While this study offers a large-scale analysis of AI health startups, it is important to acknowledge several limitations related to data sources and analytical tools. First, the proprietary nature of the PitchBook dataset limits full reproducibility, as future researchers may not have access to the identical company records, founder profiles, or investment information that were available at the time of data collection. Differences in database coverage, updates, or subscription tiers may affect the comparability of results. Second, the use of GPT-4 for large-scale classification introduces dependencies on model versioning. As LLMs evolve, future versions may yield slightly different categorizations even when given the same prompts and inputs, potentially affecting the consistency of automated coding.

Additionally, the availability and completeness of public information about founders and startup websites, used to supplement and validate PitchBook records, vary substantially across companies and over time. These factors create natural constraints on the reproducibility and stability of our findings. To support transparency, we have publicly released the code and prompts used for classification in the Online Supplementary Repository; however, full replication will inevitably depend on future access to comparable data, LLM versions, and publicly available startup information.

Looking ahead, future research can deepen our understanding of how varying levels of AI complexity shape startup capabilities, commercialization strategies, and regulatory engagement. For instance, lower-complexity systems may benefit from rapid deployment and broader applicability but face challenges in clinical legitimacy. At the same time, higher-complexity solutions require deeper partnerships, greater technical expertise, and more intensive validation. Particularly pressing is the need to examine how AI health startups balance development speed with clinical rigor across complexity tiers; how reimbursement mechanisms and institutional trust shape adoption at different levels of sophistication; and how legitimacy is constructed and maintained as AI systems evolve in autonomy and scope. These questions extend the descriptive insights of our study and invite deeper theorizing on the entrepreneurial, organizational, and systemic dimensions of AI in healthcare.

## Methods

### Sample and data

We identified a sample of AI health startups from the PitchBook database, which provides comprehensive information on companies, deals, investors, and individuals, including company identification, financial status, investment data, executive information, and valuation metrics. We began by applying two key filters available in PitchBook: the *Verticals* column and the *Primary Industry Sector*. First, we selected all ventures classified under “Artificial Intelligence” in the Verticals column. Within this subset, we filtered for startups with a Primary Industry Sector of “Healthcare.” This process resulted in an initial pool of 4,790 startups operating at the intersection of AI and healthcare.

From this pool, we retained only ventures with sufficient and reliable information to analyze founding teams. Specifically, we required complete or verifiable data on founders, including names and professional roles. For startups where founder information was missing, incomplete, or potentially mislabeled (for example, regarding founder position or gender), we manually cross-checked these details on LinkedIn. LinkedIn profiles enabled us to verify founder roles, confirm gender presentation, and review professional and educational backgrounds, thereby improving data accuracy. In a limited number of cases, Crunchbase was also consulted to resolve remaining ambiguities. After this validation step, the final sample consisted of 3807 startups for which founder-level data could be considered reliable. PitchBook remained our primary data source and defined the inclusion criteria, while LinkedIn (and occasionally Crunchbase) was used solely to confirm or complete missing or ambiguous founder information.

To enhance research transparency and ensure consistent interpretation of variables, we constructed a structured data dictionary in the form of an Excel file, which has been made publicly available through a GitHub repository. This file systematically documents the variables included in the dataset by listing each column name alongside its data type, descriptive annotations, and representative sample values. The documentation covers both the raw data fields obtained from the PitchBook database and the derived variables generated through the research team’s large language model (LLM) procedures. By explicitly specifying variable definitions and formats, this resource supports reproducibility, facilitates accurate data cleaning and analysis, and provides a shared reference framework for collaborative research.

Our analysis focuses on startups that explicitly integrate AI into healthcare-related products and services. This includes, for example, firms developing AI-driven diagnostic tools, predictive analytics, decision-support systems, and AI-enabled therapeutic technologies. To further enhance accuracy and improve data quality, information from PitchBook was supplemented with data obtained from company websites and public platforms such as LinkedIn and Crunchbase. These additional sources were used, with both manual verification and the assistance of large language models (LLMs), to update information on business ownership status (e.g., acquisitions or bankruptcies), operational focus, and core technologies. We coupled manual expert coding with LLM-assisted classification to ensure both accuracy and scalability; the manual subset grounds the taxonomy in domain expertise, while the LLM extends this logic consistently to the full dataset. The code used for the LLM analysis is shared in the Online Supplementary Material in Python code. Expert validation then reinforces reliability, reduces classification bias, and confirms alignment with established frameworks. Together, these methods provide a rigorous empirical basis for our findings, acknowledge the study’s descriptive nature, and lay the groundwork for future causal or longitudinal analyses.

A central motivation behind our sample construction is the recognition that both artificial intelligence and healthcare are highly complex domains. Building on work that conceptualizes AI as a complex adaptive system^43^, we account for differences in technological complexity across ventures. Prior research shows that the number and interdependence of knowledge components shape innovation processes, problem-solving requirements, and the probability of successful technological recombination. In a healthcare context, which is already characterized by regulatory, institutional, and professional complexity, the introduction of AI creates a “double complexity” setting. Consequently, we distinguish between ventures that rely on relatively simple analytic techniques and those that deploy more advanced AI applications, such as early imaging detection or sophisticated clinical decision-support systems. This allows us to capture better, meaningful differences in technological intensity and innovation potential among AI health startups.

In addition to technological characteristics, we incorporate geographic data to examine the spatial distribution and regional dynamics of AI health startup formation. Rather than treating location as a mere control variable, we consider it a theoretically meaningful dimension of entrepreneurial activity. Entrepreneurship and venture financing are spatially embedded, and geographic proximity to capital, talent, hospitals, and research institutions shapes both access to resources and knowledge spillovers. Prior research^64,65^, shows that local venture capital availability and the size of innovation clusters are associated with higher venture formation rates and stronger firm performance. This is particularly relevant for AI health ventures, given that regulatory regimes, data access, clinical partnerships, and reimbursement conditions are often locally bounded. Accordingly, startups were classified based on their proximity to major innovation hubs (such as Boston, San Diego, and San Francisco) and whether they remained embedded in their original regions.

Finally, we include founding team composition, particularly gender composition, as a theoretically meaningful variable. Prior work^87,98^, documents that gender diversity in entrepreneurial teams is associated with improved innovation outcomes and can strengthen the positive effects of functional diversity. This is especially relevant in AI-health ventures, which require integrating heterogeneous expertise, including clinical knowledge, data science, regulatory understanding, and entrepreneurial skills. Gender-diverse teams may also be more sensitive to differences in patient needs and potential biases in medical data and AI algorithms. We therefore treat founding team composition not as a descriptive characteristic, but as a key micro-level indicator of a venture’s capacity to integrate knowledge and innovate in complex environments.

Together, the selected variables, technological complexity, geographic embeddedness, and founding team composition, allow us to capture both structural and micro-foundational drivers of entrepreneurship in AI-enabled healthcare, and to systematically analyze heterogeneity in venture formation and development in this emergent sector.

### Methodology

Our analysis focuses on 3807 AI health startups founded between 2010 and 2024. We assessed the nature and extent of their AI capabilities by examining how AI technologies are applied across prominent medical domains and innovation areas within the health sector. To support accurate and reliable classification, we adopted a mixed-method approach combining manual coding, large language models (LLMs), and expert validation. We first identified common categories of AI-driven health applications by reviewing medical-related literature and books, industry reports, and startup profiles from platforms such as CB Insights^7,99–102^. From this review, we derived ten representative categories of AI-driven medical domains, which together form the analytical backbone of our framework and are presented in Table 6.

**Table 6:** AI medical domains, definitions, and subcategories

AI Medical Domains	Definition	Subcategories	Examples
**Clinical decision support**	Enhances medical decisions with targeted clinical knowledge, patient information, and other health data.	Patient safety, clinical management, preventative care, and healthcare logistics.	Sepsis prediction, drug-drug interaction alerts.
**Drug discovery**	Applies AI for discovering new drugs through data analysis and simulations.	Biotechnology platforms, bioinformatics, and market analysis.	AI-powered drug screening, biochemical interaction predictions.
**Imaging and diagnostics**	Uses AI for medical imaging and diagnostic processes.	Imaging (MRI, CT, ultrasound), diagnostics (ECG, laboratory diagnostics).	MRI image analysis, rapid diagnostics.
**Lifestyle and wellness**	Non-clinical technologies that aim to improve users’ lives by influencing habits through coaching, monitoring, and informing.	Fitness, nutrition, sleep monitoring, physiotherapy, and consumer health.	Fitness trackers, nutrition-coaching apps, sleep-monitoring systems, physiotherapy guidance tools.
**Medical monitoring**	Improves access to healthcare through remote monitoring and telemedicine.	General telemedicine services, telehealth platforms, specialized telemedicine (e.g., telepsychiatry).	Telecardiology, teledermatology.
**Medical treatment and personalized medicine**	Uses advanced AI for tailoring treatments to individual patients.	Multi-modal bioinformatics, regenerative medicine, and implantable devices.	Genomics-based treatment planning, tissue engineering.
**Mental health**	Uses AI to improve therapy outcomes and support mental health initiatives.	Virtual reality tools, psychoeducation, and mindfulness platforms.	Mood tracking apps, digital diaries.
**Public health and epidemiology**	Leverages AI for large-scale health monitoring and prediction.	Public health surveillance, infection prevention, and spatial modeling.	Disease forecasting, epidemic modeling.
**Rehabilitation and assistive technologies**	Helps individuals with disabilities through AI-based assistive tools.	Assistive devices, low vision aids, and environmental interaction tools.	Rehabilitation robotics, mobility aids.
**Surgery**	Enhances surgical procedures and outcomes through AI.	Acute decision support, robotic-assisted surgeries.	AI for surgical navigation and workflow optimization.

The development of this framework followed a structured, multi-stage process. First, a research assistant and the first author jointly developed the initial categorization framework using a comprehensive set of academic and practitioner references, all of which are listed in the Supplementary Materials and publicly available in the project’s online repository. This step ensured that the selected domains were theoretically grounded, empirically relevant, and representative of the diversity of AI applications in healthcare.

Once the framework and domain definitions were established, the research assistant and the first author independently conducted manual coding of a random sample of 400 startups. Each of these startups was assigned to one and only one primary medical application domain based on a close review of the company’s official website and PitchBook description. This independent, parallel coding process allowed us to assess the clarity, consistency, and applicability of the framework before scaling it to the full dataset. Disagreements in classification were resolved through discussion, leading to minor refinements of category definitions and more straightforward decision rules. This process produced the final schema shown in Table 6.

To scale the classification to the full dataset, we used the OpenAI API (GPT-4) as a large language model (LLM) to code the remaining startups, which is available in the online code. The LLM was provided with structured prompts and company descriptions extracted from PitchBook, as well as content collected from the landing pages of startup websites. The prompt-engineering code used in this process is fully transparent and available in our public GitHub repository. The LLM-based classification was then compared to the independent manual coding of the 400 startups. The classifications matched in approximately 95% of cases, with the remaining 5% involving only minor discrepancies, typically between adjacent or closely related categories. In several instances, the LLM provided more nuanced distinctions than human coders, but these suggestions were accepted only when they clearly aligned with the predefined framework.

Following this, we conducted an informal expert (“friendly”) review with three external specialists: a professor of medicine in Australia, a digital health expert in Germany, and a pharmaceutical industry expert in Switzerland. These reviewers evaluated both (a) the overall structure of the framework and (b) a subset of classified startups to verify that the categories and assignments were conceptually appropriate, empirically defensible, and clinically meaningful. Together, these steps provided an additional layer of external validation.

### Classifying and defining complexity of AI systems capabilities and architectures

We analyzed and classified startups’ AI systems by evaluating their learning architectures and technological complexity. In our LLM approach, we evaluated AI systems by assessing data uniqueness and algorithmic innovation using information from company websites and descriptions provided in PitchBook. The LLM output presented a five-tier AI systems complexity framework. We assessed our classification procedures against two primary sources of work, taxonomies formulated by leading global standards-setting and policy-guiding institutions (e.g., WEF, OECD, NIST) and the literature on digital health and medicine. We first assessed the frameworks developed by OECD^103^, NIST^104^ (e.g., AI RMF 1.0), and the World Economic Forum (WEF)^105,106^. The OECD’s Classification Framework for AI Systems (2022) provides a highly detailed, policy-oriented structure based on key system dimensions, including AI model characteristics, autonomy, task, data provenance, and human rights implications. Its purpose is to guide risk assessments, incident reporting, and regulatory policy. Similarly, the NIST’s AI Risk Management Framework^104^ focuses on risk framing and lifecycle management of AI, with an emphasis on trustworthiness (validity, reliability, transparency, explainability, etc.). The WEF’s Empowering AI Leadership Report (2022) and AI Governance Alliance report (2024) offer strategic and organizational perspectives, emphasizing responsible governance, adoption readiness, and integration of AI technologies. WEF^105^ also reported AI capability levels as “assisted,” “augmented,” and “autonomous,” which are commonly used in consulting and industry reports. While these frameworks are invaluable for regulators, risk managers, and organizational leaders, they are primarily high-level and sector-agnostic. They do not aim to classify the levels of technological complexity of AI systems per se, particularly not in domain-specific contexts such as healthcare, where technological sophistication can affect regulatory scrutiny, capital intensity, organizational capabilities, and patient risk profiles.

Next, we reviewed the literature that sits at the intersection of medical AI (clinical machine learning) and biomedical informatics/digital health, spanning foundational deep learning, clinical applications, and the translation, governance, and emerging agency of AI systems in real-world care. Several influential reviews have examined AI in medicine, each emphasizing different attributes of the models being appliedTopol (2019: 51)^7^. juxtaposed human and AI interaction in medicine using five levels of autonomous-driving vehicle analogy, focusing primarily on deep learning models with examples across six medical fields, including radiology, pathology, dermatology, ophthalmology, cardiology, and gastroenterology. Beam and Kohane^107^ mapped the landscape differently, creating a continuum of human-to-machine decision-making effort—from human agents operating through basic machine learning algorithms (e.g., regression, random forests) to deep learning models (e.g., convolutional neural networks, generative adversarial networks)—scaled against data sample size. More recently, Rajpurkar et al.^108^, extended this perspective by examining how deep learning models that require massive amounts of labeled data are being complemented by alternative paradigms, including unsupervised learning, semi-supervised learning, causal inference, and reinforcement learning. Each of these reviews offers valuable framing, highlighting different attributes—autonomy level, human-versus-machine decision effort, and learning paradigm. Much of the broader literature, however, has adopted more simplistic, dichotomous labels when classifying AI systems in healthcare. Common binary framings include: “knowledge-based versus non-knowledge-based” clinical decision support by Sutton et al.^109^; “deterministic versus adaptive” systems by Cohen^28^; and “nongenerative predictive analytics versus generative AI” by Rashidi et al.^110^. While each of these dichotomies captures a real distinction, they flatten the considerable heterogeneity that exists among AI systems in terms of architectural complexity, learning capability, data requirements, and clinical integration pathways.

The American Medical Association (AMA) has also proposed a taxonomy classifying AI applications for medical services and procedures into three categories, namely, assistive, augmentative, and autonomous, based on the degree of machine work performed relative to the clinician^111^. Although primarily designed to standardize billing and reimbursement coding in the United States, its categorical distinctions have been progressively operationalised across Current Procedural Terminology (CPT) codes for AI-enabled cardiac, imaging, and procedural services. While this represents a meaningful step toward institutional standardization, the three-tier structure was designed to answer a reimbursement question rather than to characterize the architectural complexity or learning capability of AI systems.

A comprehensive understanding of AI development requires a systematic analysis of its evolving computational paradigms and architectures, and the innovative approaches adopted by pioneering AI health startups in various medical fields. Without such examination, interpretations of AI progress remain restricted, especially considering that systems classified under a common designation may exhibit significant differences. For instance, a simple logistic regression model predicting hospital readmission and a multi-modal transformer integrating genomic, imaging, and clinical data to recommend personalized therapies are both “predictive,” yet they differ fundamentally in architecture, data complexity, explainability, and regulatory pathway. Likewise, a rule-based chatbot answering patient FAQs and a large language model generating clinical documentation are both “generative,” yet operate under entirely different computational paradigms. Our five-tier framework addresses this gap by placing AI systems on an ordinal scale of architectural complexity. This matters for two reasons. First, the level of technical complexity directly shapes the regulatory, clinical, and organizational challenges associated with deployment^28,29^. Second, disaggregating AI systems by complexity level allows us to examine whether the patterns we observe in startup founding, investment, and clinical adoption differ systematically across tiers—an analytical dimension that requires a more refined AI systems complexity framework.

Building on our review of these broader AI frameworks, medical literature, and the AI health applications developed by the AI health startups in our dataset, we found their AI health innovations most suitably mapped onto these levels labeled as Deterministic AI, Assistive AI, Perceptual AI, Integrative AI, and Autonomous-Agentic AI, as shown in Table 7. Our five-tier framework was developed to extend our understanding of AI use in healthcare by analytically categorizing AI health startups based on the technological complexity of their core AI systems, ranging from basic rule-based engines to pioneering, adaptive, multi-source models operating with near-autonomous decision-making. This complements the aforementioned frameworks by offering: (1) A use-case-specific classification that captures heterogeneity in technological development across thousands of health-focused startups, (2) A continuum-based approach that reflects real-world evolution of AI sophistication (e.g., startups progressing from rule-based systems to complex deep learning models within 12–18 months), and (3) An analytical lens on innovation outcomes, such as how increasing AI complexity may impact business model scalability, regulatory compliance burdens, data infrastructure needs, and investor expectations.

**Table 7:** Five-tier AI systems complexity framework

Level	AI systems label	Definition	Examples of adoption in healthcare	AI models+ training sys
1	**Deterministic AI** (Low Complexity)	Rule-based or symbolic systems that follow fixed “if–then” logic without learning or adapting.	▪ Appointment scheduling tools using logic trees ▪ Clinical decision support alerts for drug–drug interactions in EHRs ▪ Triage algorithms in urgent care kiosks	None (no ML)
2	**Assistive AI** (Moderate Complexity)	Basic machine learning models that support decisions on structured data and require frequent human input or supervision.	▪ Chatbots answering FAQs (e.g., Ada Health) ▪ Early clinical coding and billing automation ▪ Logistic regression models predicting readmission risk	FNN, MLP
3	**Perceptual AI** (High Complexity)	Deep learning systems capable of interpreting unstructured data like images, audio, or free-text medical records.	▪ Radiology AI tools detecting fractures, nodules, or pneumonia on X-rays (e.g., Aidoc, Zebra Medical Vision) ▪ Dermatology apps detecting skin lesions (e.g., SkinVision)	CNN, RNN
4	**Integrative AI** (Advanced Complexity)	Multi-modal systems that combine and analyze data from multiple sources (genomic, clinical, lifestyle) with minimal human oversight.	▪ Oncology platforms combining imaging, biopsy, and genomics to recommend personalized therapies (e.g., Tempus) ▪ Chronic disease AI coaches integrating EHR + wearables data	C/G/RNN TRF
5	**Autonomous- Agentic AI** (Pioneering Complexity)	Autonomous AI systems operating within tightly defined clinical tasks to broader agentic systems capable of multi-step reasoning, adaptive decision-making, autonomous action-taking, and continuous learning across distributed clinical sites with minimal human intervention.	▪ IDx-DR was the first autonomous AI diagnostic system to receive FDA clearance across any field of medicin, with a dedicated CPT billing code subsequently established for autonomous AI diagnostic devices	All included

*FNN* Feedforward neural networks, *MLP* Multi-layer Perceptrons, *CNN* Convolutional Neural Networks, *TRF* Transformer Models, *GNN* Graph Neural Networks, *ML* Machine Learning, *RNN* Recurrent Neural Networks,

Centralized Learning (a hub),  Decentralized Learning (a network),  Federated Learning (a global cloud with local hubs),  Edge Learning (a global hub with local devices)

Table 7 categorizes healthcare AI systems into five levels of increasing complexity, from Deterministic AI (rule-based systems with no machine learning) to Autonomous-Agentic AI (systems capable of real-time, continuous learning). Each level is defined by the system’s learning capacity, adaptability, and level of integration with data sources, and is illustrated with healthcare-specific examples. At higher complexity levels, systems increasingly rely on advanced neural network architectures such as Transformers (TRF) and Graph Neural Networks (GNN), and may require multimodal data integration or distributed training infrastructures such as federated learning. We provide an online supplementary Appendix on GitHub that includes a summary of the literature we use to define all the technical labels as seen in Table 7.

To assign individual startups to these tiers, we relied solely on publicly available information, including content on company websites, landing pages, and descriptions in the PitchBook database. Importantly, we did not infer or assume any technical features that the startup did not explicitly state. If information was not available online, the company was coded solely using the PitchBook description. Next, a consistent rule-based mapping was used to assign each startup to one of the five complexity tiers. Firms using only rules or linear regressions were assigned to Tier 1. Startups utilizing classical machine learning or simple NLP tools were assigned to Tier 2. Those employing deep learning on unstructured data were placed in Tier 3. Firms describing multimodal AI, transfer learning, or generative systems (e.g., GANs) were placed in Tier 4. Only startups explicitly referencing advanced agentic AI model or training systems such as federated learning, neurosymbolic AI, or real-time adaptive/edge systems were coded as Tier 5. This rules-based approach minimized subjectivity and ensured consistency.

Finally, to ensure transparency and replicability, we provide a detailed coding appendix. This includes the complete rule document (“AI Complexity classification.pdf”), examples illustrating how real startups were classified, an explanation of borderline or ambiguous cases, and the complete prompt-engineering code used for LLM classification. These materials are publicly available through our GitHub repository.

## Supplementary information

Supplementary material

## Data Availability

A sample of data used in this work is available in a public GitHub repository at https://github.com/MrGluten/LLM-category-complexity.

## Code availability

The custom code used for automated classification of healthcare startups in this study is publicly available at https://github.com/MrGluten/LLM-category-complexity under an MIT License with no restrictions on access.
