# Supplementary material for: Mapping AI startup investment and innovation in healthcare using a five-tier AI systems complexity framework
Source: NPJ Digit Med. 2026 Apr 14;9:458. doi: 10.1038/s41746-026-02595-5 (PMC13269711; doi:10.1038/s41746-026-02595-5)
Supplement: Supplementary file 1 — Supplementary material [file 41746_2026_2595_MOESM1_ESM.pdf]

Supplementary Information: AI in Healthcare Categorization Framework

This supplementary document provides a comprehensive framework for categorizing and classifying AI advancement in healthcare technologies. It contains detailed taxonomies across multiple dimensions:

Medical Field Categories: The document outlines nine major healthcare domains where AI is being applied, including Clinical Decision Support, Imaging and Diagnostics, Drug Discovery, Mental Health, Surgery, Medical Treatment and Personalized Medicine, Public Health and Epidemiology, Medical Monitoring, and Rehabilitation & Assistive Technologies. Each category includes specific application-based or technology-based subcategories.

AI Technology Classification: It defines four main technology types used in healthcare AI: Machine Learning, Computer Vision, Natural Language Processing, and Bayesian Predictive Modeling.

AI Complexity Framework: The document presents a five-level complexity scale for evaluating AI implementation:

- Low Complexity: Rule-based systems with minimal autonomy
- Moderate Complexity: Basic machine learning with supervised learning
- High Complexity: Deep learning and neural networks
- Advanced Complexity: Transfer learning and multimodal AI
- Pioneering Complexity: Federated learning and neurosymbolic AI

Evaluation Dimensions: It includes frameworks for assessing AI systems based on autonomy levels (Perception, Decision, Action) and impact levels (Low, Medium, High), providing guidance for analyzing healthcare AI startups and technologies.

The document serves as a reference guide for systematically evaluating and categorizing AI technologies in the healthcare sector.

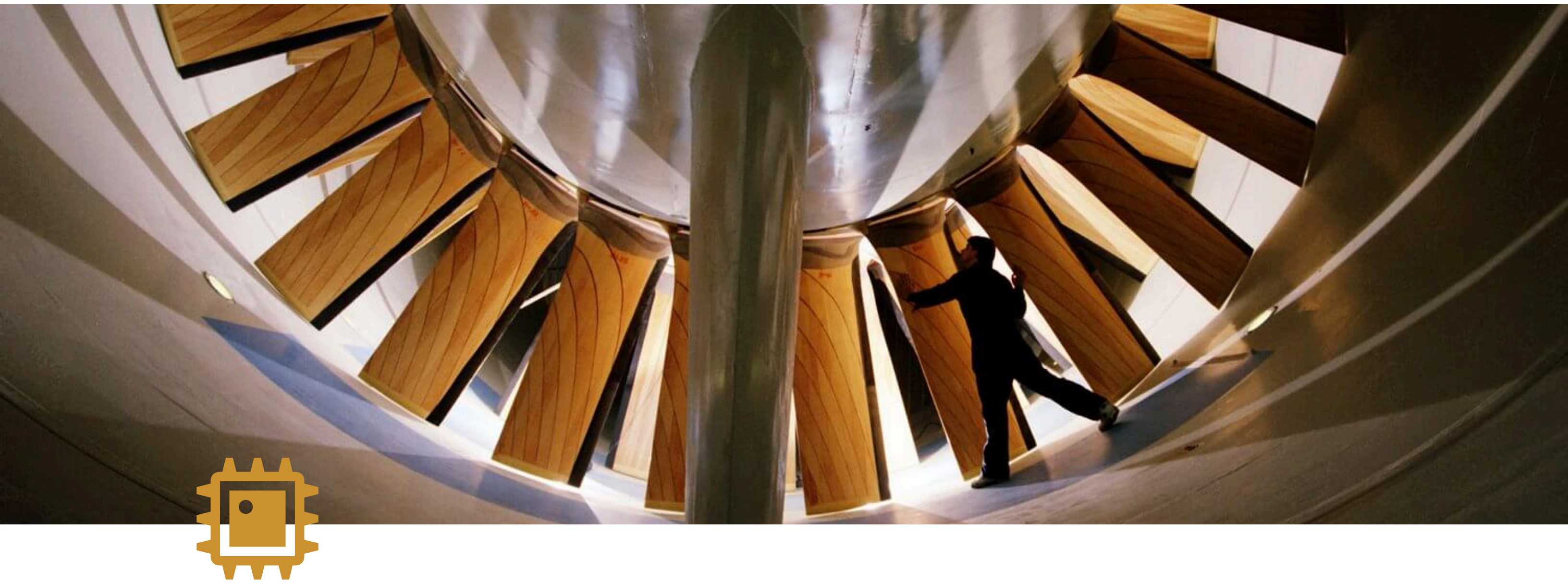

Categorization of Medical Fields & Classification AI Advancement

New AI in Healthcare Categorization

📌 Categorization according to use case of AI

▼ ✅ Clinical Decision Support

www.nature.com

https://www.nature.com/articles/s41746-020-0221-y

An overview of clinical decision support systems: benefits, risks, and strategies for success

Advanced integration of Electronic Health Record (EHR)

> A clinical decision support system (CDSS) is intended to improve healthcare delivery by enhancing medical decisions with targeted clinical knowledge, patient information, and other health information.
> Osheroff, J. et al. *Improving Outcomes with Clinical Decision Support: An Implementer's Guide*. (HIMSS Publishing, 2012).

How to Classify the AI Advancement of the technology?

Difference between knowledge based (IF/THEN) and non-knowledge based (AI Inference)

> A common differentiation is between knowledge-based (IF/THEN) and non-knowledge-based (AI/ML) CDSS'. For simplicity: purely knowledge-based CDSS will be classified between Low and Moderate, whereas non-knowledge based CDSS will be classified between Advanced and Pioneering. The category High will be used if the technology uses multiple modalities for their ML model, but isn't truly non-knowledge based.

Application based subcategories

- **Patient Safety**
  - Drug Control
    - Medication administration errors
    - Drug-drug Interactions (DDI)
  - Hygiene Compliance
  - Sepsis Prediction

The potential of artificial intelligence to impr…
npj Digital Medicine - The potential of artificial intelligence to improve patient safety: a scoping
https://www.nature.com/articles/s41746-021-0…

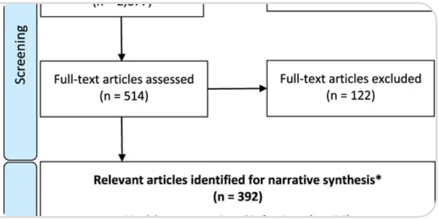

- **Clinical Management**
  - Clinical Trial Technology Personalized
    - Finding eligible patients
  - Clinician guideline adherence
  - Patient Management
    - Research/Treatment protocols
  - Healthcare Logistics
    - Tracking and Placing Orders
  - Follow-up referrals
  - Preventative Care
- **Cost Containment**
  - Decreasing patient length-of-stay
  - Clinical Interventions
  - Payment Operations
  - CPOE-integrated systems for suggesting cheaper medication alternatives
  - Reducing test duplication

- **Administrative Functions**
  - Clinical Workflow Tools
  - Documentation Templates
  - Patient Triage
- **Diagnostics Support**
  - Imaging
  - Laboratory and Pathology
  - Virtual Assistant
- **Health Data Interoperability and Integration**
  - Secure Data Exchange
  - Health Data Platforms

Imaging and Diagnostics

💡 The imaging and diagnostics technologies are the end itself.

Technology based subcategories

- Diagnostic Devices
  - ML Enhanced Hardware, e.g. stethoscope
  - ECG
  - EEG
- Diagnostic Imaging Software

  Imaging Technologies:

  - Ultrasound
  - Magnetic Resonance Imaging (MRI)
  - Computed Tomography (CT)
- Biological Diagnostics
  - Rapid Diagnostics
  - Laboratory Diagnostics
    - PCR
    - Bacterial cultures
- Digital Biomarkers

  Technologies used

  - Speech-based
  - Passive Sensing
  - Active Participation based Digital Biomarkers

Drug Discovery

💡 Applying AI in the discovery of new drugs. Be it by advance drug target screening, biochemical interactions, in silico trials. Special Highlight on the financial investments into this sector by big Pharma.

Technology based subcategories

- Biotechnology Platform
- Bioinformatics
- Market Analysis (**Application based**)

✅ Mental Health

💡 Using AI in remote and in-person settings to improve therapy outcomes. IMPORTANT: Healthcare provider must be in the loop, otherwise the company will be classified as a Lifestyle and Wellness company, which does not fall under Digital Medicine (i.e. Healthcare).

Digital transformation of mental health services

npj Mental Health Research - Digital transformation of mental health services

https://www.nature.com/articles/s44184-023-00033-y

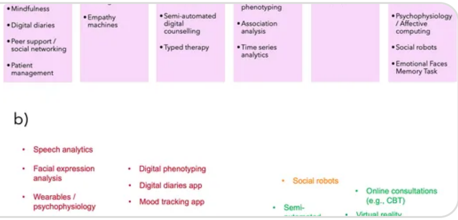

Digital transformation of mental health services

Application based subcategories

Digital Health Apps

- Mood Tracking
- Psychoeducation
- Mindfulness
- Digital Diaries
- Peer Support
- Social Networking
- Patient management

Surgery

💡 Improving Surgeries through the use of AI.

Application based subcategories

- Surgical Navigation
- Acute Decision Support
- Workflow Optimization

Medical Treatment and Personalized Medicine

💡 Using advanced technologies in genomics, biomedical engineering, robotics and sensing technologies to personalize the treatment of the patient.

Technology based subcategories

- Genomics
  - Genomics
  - Proteomics
  - Epigenomics
  - Multi-omics
- Biomedical Devices
  - Implantable Devices
  - Non-invasive stimulation therapeutic devices
- Regenerative Medicine (**Application based**)
  - Tissue Engineering
- Reproductive Medicine (**Application based**)
  - In vitro fertilization technologies

✅ Public Health and Epidemiology

💡 Using AI to understand and impact the big picture in healthcare.

Frontiers | Using artificial intelligence to improve p...

Artificial intelligence (AI) is a rapidly evolving tool revolutionizing many aspects of healthcare. AI has been

https://www.frontiersin.org/journals/public-health/artic...

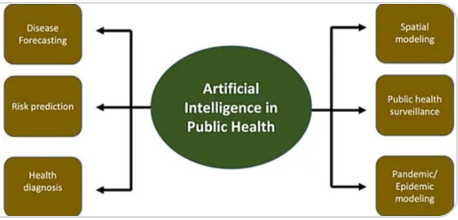

Application based subcategories

- Disease Forecasting
- Risk Prediction
- Health Diagnosis
- Spatial Modeling
- Public Health Surveillance
- Epidemic Modeling
- Infection Prevention and Control

Medical Monitoring

💡 Using AI to improve access to healthcare using purely remote healthcare delivery.

Application based subcategories

- Telemedicine
  - General Telemedicine Services
  - Specialized Treatment in Telemedicine
  - Telehealth Platforms and Tools
  - Telerehabilitation
- Digital Biomarkers Monitoring
  - Biomarkers extracted through passive sensing from mobile phones or smartwatches
- Monitoring Devices (Technology based)

  Examples:

  Bra monitoring heart health (bloomer health tech)

Using above review, named: **Review of Systematic Reviews in the Field of Telemedicine.** *Categories adapted from it.*

💡 Last Point before lunch! Adapt all Categories mentioning Wearables

---

✅ Rehabilitation & Assistive Technologies

Review of Recent Research Trends in Assistive Tec...

Globally, there is more than half a billion disabled people due to physical, mental, or sensory deficiencies.

https://ieeexplore.ieee.org/document/8925327

Application based subcategories

Assistive Devices (ATs)

- Survival
- Communication
  - Low Vision Aid
- Environmental Interaction
- Mobility
- Physical Education
- Positioning

**'s for Survival** tain life including, bathing aids, feeding, of these technologies include aids for daily d cutlery (e.g. jar opener, potato peeler), th seat, bath or toilet hoist, ripple mattress, ok, and put on/off clothing.	**ATs for M** Tools and devices to assist people with m navigation in indoor and outdoor environ opportunity to navigate their home and walk, climb stairs, and transfer from whe technologies include wheelchairs, exoskel
**r Communication** or social interaction, visual and auditory iculation. Examples of such technologies hesizers, low-vision aid, magnifying glass, , Braille display and writer, flashing light arm clocks.	**ATs for Physica** Tools designed to empower persons with physical exercises. There are numerous which includes adapted wheelchairs and a persons to take an interest in swimmi recreational exercises.
**ironmental Interaction** eople with disabilities to interact with their l devices, electronic doors, foot mouse for nd functions, adapted light switches, door ramps, handrails, and bath grab-rail.	**ATs for Pos** These ATs meet the needs of individua cannot support themselves in positions su down. Such ATs include adaptive seat tables, standing frames, bean bag chairs, a

---

Fall out categories

CDS

Patient-facing decision support Participatory

- Patient controls EHR data

Technology Column

4 categories will be used, which are the following:

- Machine Learning
- Computer Vision
  - Image recognition and classification
  - Object detection
  - Object tracking
  - Facial recognition
  - Content-based image retrieval

  Source below:

  Types of Artificial Intelligence | IBM
  Early iterations of the AI applications we interact with most today were built on
  https://www.ibm.com/think/topics/a...
- Natural Language Processing
- Bayesian Predictive Modeling

Judging AI Advancement

Each category will have a slightly different approach. Let's provide a general framework.

Given that currently the vast majority of

Questions to filter:

1. Do they have an in-house data science team or are they using their models from third parties?
   a. if not clearly findable, look for keyword matching, e.g. proprietary, own model, finetuning → inhouse is higher level implementation, outsourcing lower.
2. Compare inhouse/outsource to funding of the startup (idea).

Evaluate the governance style of the company, is it centralized or decentralized?

- Breaking down the AI implementation into measurable dimensions. Following examples:
  - inhouse/outsource
  - what kind of models are they using? complexity level of these models.
  - List

🤖 Categories for Technologies used

Fall Out Categories

▼ Virtual Assistant

▼ Lifestyle & Wellness

(Non-)clinical technologies that aim to improve the user's life by influencing habits, be it through coaching, monitoring, informing or other means.

- ▶ Fitness
- ▶ Nutrition
- ▶ Sleep Monitoring
- ▶ Physiotherapy
- ▼ Consumer Health

  Consumer Health refers to the active participation of individuals in making informed decisions about their own health or the health of their loved ones, often by accessing and evaluating health information available online.

  www.sciencedirect.com

  https://www.sciencedirect.com/topics/social-sciences/consumer-health

  Reference to Call-out (reference AI generated)

- Virtual Assistant (Telemedicine)
  - Disease Management
  - Monitoring
  - Consultation
  - Triage and Appointment Making
  - Diagnosis
  - Clinical Care
  - Follow-Up
  - Medical Education or Training
  - Clinical Trial Support
  - Knowledge Dissemination
  - Drug Delivery

Review on Chinese Telemedicine

Implications for implementation and adoption of tel…

npj Digital Medicine - Implications for implementation and adoption of telehealth in developing countries: a systematic

https://www.nature.com/articles/s41746-023-00908-…

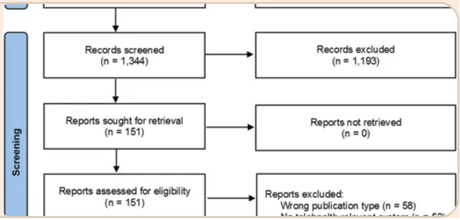

Medical Domains

Can be very many different ones. Focus on the existing medical domain list as in the following link:

Specialty Profiles | Careers in Medicine

Browse the profiles of the more than 160 specialties in the United States and nearly 40

[illegible]

How to calculate a (rough) score of AI integration and maturity in these startups?

Figure out a system by calculating some score from scratch. What kind of algorithms is the company using? For

[illegible]

Classify AI Implementation

▼ Current Framework on AI Complexity

What she did in the report is give people to try the algorithms and assess the complexity based on different elements that are: ——————————- Autonomy and Impact

And then we complete this work to build this five scale point complexity framework.

1. Low Complexity:

Description: AI systems in this category rely heavily on predefined rules, decision trees, or simple statistical models. They perform tasks that are well-structured, with limited variability, and typically don't evolve or learn from new data over time.
Technologies:
Rule-based systems: These systems operate on clear "if-then" logic. There is no learning involved, and all decisions are based on static rules set by developers.
Simple regression models: Linear or logistic regression models that work well with small, structured datasets and don't require advanced computational power.
Characteristics:
Minimal or no autonomy: Human intervention is needed to define parameters, interpret results, and modify the system.
Structured data: Operates on clean, labeled datasets.
Examples: Administrative automation (e.g., appointment scheduling), simple clinical decision support systems that follow predefined clinical guidelines.
2. Moderate Complexity:

Description: Systems in this category incorporate basic machine learning techniques that involve some level of training and adaptation. These models

are still highly reliant on structured data but exhibit improved pattern recognition capabilities.
Technologies:
Supervised learning models: Algorithms like support vector machines (SVMs), decision trees, and random forests. These models can generalize patterns from labeled data but require consistent supervision and tuning.
NLP models for structured text: Basic natural language processing for text classification and sentiment analysis.
Characteristics:
Limited autonomy: These models need to be re-trained regularly and may require human intervention to handle outlier cases or novel inputs.
Moderate complexity in handling structured data, with some capability to generalize.
Examples: Diagnostic tools for analyzing radiology reports, basic chatbots that provide information based on predefined responses.
3. High Complexity:

Description: Systems at this level utilize deep learning or neural networks capable of working with large datasets and unstructured data, such as medical images or genomic data. These systems are often applied in healthcare for more complex tasks such as medical imaging analysis or predicting disease progression.
Technologies:
Convolutional Neural Networks (CNNs): Commonly used in computer vision applications for image analysis in radiology, dermatology, or pathology.
Recurrent Neural Networks (RNNs): Used for time-series data or longitudinal patient records, especially in monitoring patient vitals or disease trajectories.
Characteristics:
Moderate to high autonomy: These models can adapt to new data but may require periodic re-training and validation by human experts.
Ability to handle unstructured data: These models can process and make sense of complex, high-dimensional data such as MRI scans or EHR records.
Examples: AI-based diagnostics (e.g., detecting tumors in MRI scans), AI for personalized medicine using patient history and genomics.
4. Advanced Complexity:

Description: Advanced AI systems use techniques like transfer learning, multimodal AI, or reinforcement learning. These systems can combine disparate data sources (e.g., text, images, and clinical notes) and adaptively improve their performance with little human intervention.
Technologies:
Transfer Learning: This allows models to leverage knowledge from one domain to improve performance in another domain, reducing the need for extensive training on new tasks.
Generative Adversarial Networks (GANs): Often used to generate synthetic medical images or simulate patient outcomes.
Characteristics:
High autonomy: Systems can operate with minimal human intervention and adapt quickly to new data inputs.
Handles both structured and unstructured data: These models excel at integrating data from multiple sources to make comprehensive predictions or assessments.
Examples: Personalized treatment planning combining genetic data, medical history, and lifestyle factors, advanced surgical assistance systems that provide real-time feedback during operations.
5. Pioneering Complexity:

Description: These systems represent the cutting-edge of AI technologies and include neurosymbolic AI, federated learning, and edge AI. They are often deployed in environments where real-time decision-making and adaptive learning are critical.
Technologies:
Federated Learning: AI models are trained across decentralized data sources (e.g., hospitals), improving privacy and scalability while leveraging large-scale data without needing centralization.
Neurosymbolic AI: Integrates symbolic reasoning with deep learning to create models that can reason more abstractly and handle more diverse cognitive tasks.
Characteristics:
Full autonomy: Systems are capable of real-time decision-making and can adapt without human intervention.
Ability to operate in low-latency, distributed environments: These systems can

work with real-time data streams in critical care settings.
Examples: AI systems in ICUs that predict patient deterioration in real-time, federated AI systems for global pandemic monitoring and response.w

▼ Prompt for LLM Analysis of Healthcare AI Startups Using Framework C:

Analyze the provided content about healthcare AI startups, which includes a short description and, if available, a landing page. Classify each startup according to **two dimensions**:

1. **Autonomy**: Determine the level of autonomy exhibited by the AI system used by the startup:
    - **Action Autonomy**: The system operates independently, processing inputs, making decisions, and taking actions without human intervention (e.g., fully autonomous diagnostic or treatment systems).
    - **Decision Autonomy**: The system processes inputs and provides decision outputs (e.g., recommendations or predictions), but a human must act based on these outputs (e.g., clinical decision support tools).
    - **Perception Autonomy**: The system processes inputs and flags information that requires human evaluation or further action (e.g., AI used to detect anomalies in medical images for doctors to review).
2. **Impact**: Assess the potential impact of the AI system based on its risk level and influence on healthcare outcomes:
    - **High Impact**: The system's actions or decisions could significantly affect patient safety, medical outcomes, or the functioning of critical healthcare systems (e.g., AI systems used in emergency interventions or high-risk surgeries).
    - **Medium Impact**: The system influences clinical decisions, patient care, or operational efficiency but presents mitigable risks (e.g., AI for optimizing hospital workflows or assisting in routine diagnostics).
    - **Low Impact**: The system has minimal risk and may affect non-critical aspects of healthcare (e.g., AI used for patient scheduling or administrative tasks).

For each startup, classify its system into one of the three levels for both **autonomy** and **impact** based on the provided information. If only partial information is available (e.g., no landing page), use the short description to make a classification. Additionally, provide a rationale for your classification.

Based on the CSET Report, Framework C

https://cset.georgetown.edu/publication/classifying-ai-systems/
